# miR-486-5p protects against rat ischemic kidney injury and prevents the transition to chronic kidney disease and vascular dysfunction

**DOI:** 10.1042/CS20231752

**Published:** 2024-05-22

**Authors:** Adrianna Douvris, Jose L. Viñas, Alexey Gutsol, Joseph Zimpelmann, Dylan Burger, Kevin D. Burns

**Affiliations:** 1 Division of Nephrology, Department of Medicine and Kidney Research Centre, Ottawa Hospital Research Institute, University of Ottawa and the Ottawa Hospital, Ottawa, Canada; 2 Department of Cellular and Molecular Medicine, University of Ottawa, Ottawa, Canada

**Keywords:** acute kidney injury, endothelial function, ischaemia-reperfusion injury, microRNA, vasculature

## Abstract

**Aim:** Acute kidney injury (AKI) increases the risk for progressive chronic kidney disease (CKD). MicroRNA (miR)-486-5p protects against kidney ischemia–reperfusion (IR) injury in mice, although its long-term effects on the vasculature and development of CKD are unknown. We studied whether miR-486-5p would prevent the AKI to CKD transition in rat, and affect vascular function. **Methods:** Adult male rats were subjected to bilateral kidney IR followed by i.v. injection of liposomal-packaged miR-486-5p (0.5 mg/kg). Kidney function and histologic injury were assessed after 24 h and 10 weeks. Kidney endothelial protein levels were measured by immunoblot and immunofluorescence, and mesenteric artery reactivity was determined by wire myography. **Results:** In rats with IR, miR-486-5p blocked kidney endothelial cell increases in intercellular adhesion molecule-1 (ICAM-1), reduced neutrophil infiltration and histologic injury, and normalized plasma creatinine (*P*<0.001). However, miR-486-5p attenuated IR-induced kidney endothelial nitric oxide synthase (eNOS) expression (*P*<0.05). At 10 weeks, kidneys from rats with IR alone had decreased peritubular capillary density and increased interstitial collagen deposition (*P*<0.0001), and mesenteric arteries showed impaired endothelium-dependent vasorelaxation (*P*<0.001). These changes were inhibited by miR-486-5p. Delayed miR-486-5p administration (96 h, 3 weeks after IR) had no impact on kidney fibrosis, capillary density, or endothelial function. **Conclusion:** In rats, administration of miR-486-5p early after kidney IR prevents injury, and protects against CKD development and systemic endothelial dysfunction. These protective effects are associated with inhibition of endothelial ICAM-1 and occur despite reduction in eNOS. miR-486-5p holds promise for the prevention of ischemic AKI and its complications.

## Introduction

Acute kidney injury (AKI) affects up to 20% of hospitalized patients and confers a high mortality risk [1]. Patients who recover are at increased risk of progressive chronic kidney disease (CKD) [2]. An important cause of AKI is ischemia–reperfusion (IR) injury, characterized by acute tubular injury, necrosis/apoptosis, and endothelial cell injury [3]. Recovery involves repair and regeneration of tubular epithelial cells, although this process can be incomplete, resulting in interstitial fibrosis and tubular atrophy [4]. Further, in kidney IR injury, endothelial damage alters vascular reactivity [5,6], and increases the surface expression of adhesion molecules such as intercellular adhesion molecule-1 (ICAM-1) [7], thereby compromising capillary barrier function [8], and driving inflammatory cell infiltration [7,9,10]. Endothelial injury results in capillary loss, exacerbating chronic hypoxia and propagating tubular injury. These changes contribute to the transition to CKD [11–13], marked by tubulointerstitial fibrosis.

Treatment options for AKI remain limited, and microRNAs (miRNAs) have emerged as potential novel therapeutics. miRNAas are short non-coding RNAs that potently regulate gene expression by binding the 3′-untranslated region (UTR) of target messenger RNAs (mRNAs) to induce degradation or inhibit translation [14]. A systematic review identified 42 miRNA species as therapeutic targets in rodent models of AKI [15]. We have shown that one of these miRNAs, miR-486-5p, protects against kidney IR injury in mice, associated with inhibition of apoptosis and decreased kidney expression of the miR-486-5p target gene *phosphatase and tensin homolog* (PTEN) [16,17]. miR-486-5p also significantly down-regulated proximal tubular activation of genes involved in apoptosis and tumor necrosis factor (TNF)-α signaling [17].

Despite these findings, the effect of miR-486-5p on the transition to CKD remains unknown. In this regard, persistent PTEN loss in proximal tubules following IR injury may cause dysfunctional tubule regeneration and fibrosis [18], conferring a negative impact on recovery from AKI. Long-term effects of miR-486-5p on the vasculature are also unclear. In a pre-clinical study of myocardial infarction, exosomal transfer of miR-486-5p increased vascular endothelial growth factor (VEGFA) signaling and enhanced cardiac angiogenesis [19]. Exosomal miR-486-5p promoted microvascular cell proliferation, migration and angiogenesis after cutaneous wound healing, by targeting the transcriptional repressor *Sp5* [20]. In contrast, miR-486-5p was found to inhibit sprouting angiogenesis in cultured endothelial cells [21].

The uncertainty regarding potential adverse effects of miR-486-5p on long-term kidney and vascular function prompted the current studies, in which we first determined if miR-486-5p administered at the start of reperfusion would protect against ischemic AKI in rat, and then studied the impact on late development of peritubular capillary rarefaction and kidney fibrosis. We also tested if delayed administration of miR-486-5p after IR would impact peritubular capillary density, development of kidney fibrosis, and systemic endothelium-dependent vascular function.

## Materials and methods

### Kidney IR injury

**Figure 1:**
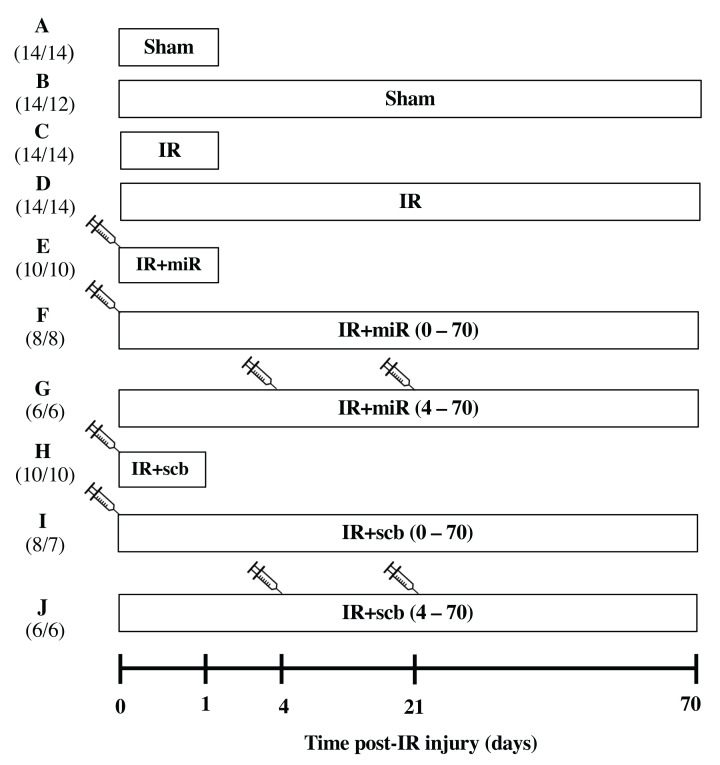
Experimental timeline illustrating study groups Rats were subjected to sham surgery or bilateral kidney ischemia-reperfusion (IR) for 45 min (time 0). End-points were set at 24 h (1 day) or 10 weeks (70 days) after reperfusion. Groups E, F, H and I received a single dose of miR-486-5p (0.5 mg/kg, IR+miR-486-5p) or scramble miRNA (0.5 mg/kg, IR+scb) by tail vein injection at the start of reperfusion. Groups G and J received two doses of miR-486-5p or scramble miRNA: the first dose on day 4 and second dose on day 21 of reperfusion. To the left of the figure, the number of animals in each group is shown in parentheses. The first number refers to the starting number of rats in each group while the second number indicates the rats that completed the study. Not shown in this timeline: a separate group of 10 rats received delayed miR-486-5p injection at 4 days after IR and were sacrificed at 5 days post-IR (group numbers included *n*=3 (sham), *n*=4 (IR), *n*=3 (IR+miR-486-5p)).

Male Sprague Dawley rats aged 6–8 weeks, weighing 250–300 g, were purchased from Charles River Laboratories (Senneville, QC, Canada) and housed at the University of Ottawa Animal Care Facility. All animal experiments were conducted at the University of Ottawa. Animal protocols were approved by the Animal Care Ethics Committee at the University of Ottawa and performed according to the recommendations of the Canadian Council for Animal Care. All rats were anesthetized with inhaled isoflurane. Briefly, the vaporizer was set to 4% then 2% isoflurane at 1.5 litres per minute for induction and maintenance of anesthesia, respectively. There was no significant difference in the duration of anesthesia amongst all groups. Bilateral flank incisions were performed, followed by bilateral renal artery clamping for 45 min. Lipid-encapsulated miR-486-5p mimic (Invivofectamine 3.0, Thermo Fisher Scientific, Waltham, MA, U.S.A.) or scramble (scb) miRNA (Thermo Fisher Scientific) was administered as a single dose of 0.5 mg/kg by tail vein injection at the start of reperfusion [17], or as delayed administration of two doses 96 h and 3 weeks after reperfusion. Sham rats underwent surgery without renal artery clamping. The number of rats ranged from 6 to 14 per experimental group. Twenty-four hours and ten weeks after reperfusion, rats were anesthetized with inhaled isoflurane for kidney blood flow measurements followed by euthanasia by decapitation (while anesthetized with isoflurane) for blood and tissue collection. Figure 1 provides a timeline schematic of the experimental protocol including number of rats used and mortality rate (3/114 rats, 2.6%).

### Regional kidney blood flow

At 24 h or 10 weeks after surgery, rats were anesthetized with inhaled isoflurane, and placed in the right lateral decubitus position. A left flank incision exposed the left kidney, which was decapsulated, and regional blood flow was measured by laser doppler flowmetry (ABLPHN20 20G probe, Transonic Scisense Inc, London, ON, Canada) with the laser doppler probe mounted onto a micromanipulator attached to a stereotaxic frame. Baseline cortical blood flow was measured by resting the probe on the kidney surface and baseline medullary blood flow was measured by inserting the probe to a depth of 3.0–4.5 mm (measured with the frame) into the kidney parenchyma [22]. Probe placement within the medulla was verified at the end by dissection. Results are reported as absolute values of tissue perfusion (arbitrary units).

### Biochemistry

Blood samples were collected in heparinized tubes for plasma separation. Plasma creatinine (Cr) and blood urea nitrogen (BUN) were measured by IDEXX Laboratories (Toronto, ON, Canada) [17]. Urine albumin and creatinines were measured by rat albumin ELISA (Nephrat II) and Creatinine Companion assay (both from Ethos Biosciences, Logan Township, NJ, U.S.A.).

### Real-time PCR

Total RNA was isolated from whole kidney, liver, heart, and spleen using the miRNeasy micro kit (Qiagen Inc., Toronto, ON, Canada). Reverse transcription and real-time PCR for miR-486-5p was performed via TaqMan™ MicroRNA Assay (Life Technologies Inc, Toronto, ON, Canada) with the Applied Biosystems 7300 real-time PCR system (Foster City, CA, U.S.A.). Endogenous U6 snRNA was used for normalization, and the relative levels of miR-486-5p were calculated using the 2^−∆∆Ct^ method [23].

### Histology

Kidneys were fixed in 3% paraformaldehyde and embedded in paraffin. All histological analyses were performed in a blinded manner by a pathologist (co-author A.G.). Tubular injury was detected by periodic acid Schiff stain, and semi-quantified using a scoring system (0-4) as described [24]. Apoptosis was measured by terminal deoxynucleotidyl transferase-mediated dUTP nick-end labeling (TUNEL) assay (ApopTag Plus Kit, MilliporeSigma Canada Ltd, Etobicoke, ON, Canada), as described [17], with counting of stained nuclei. Collagen was detected by picrosirius red staining as the percentage of collagen relative to the total area.

ICAM-1 (mouse monoclonal, Abcam Inc, Toronto, ON, Canada), endothelial nitric oxide synthase (eNOS, rabbit monoclonal, Abcam Inc), CD31 (goat anti-CD31, R&D Systems Inc., Burlington, ON, Canada), and α-smooth muscle actin (mouse α-SMA, Santa Cruz Biotechnology, Inc., Dallas, TX, U.S.A.) were detected by immunofluorescence on frozen cryostat sections (OCT embedded, 10 µm thickness). For quantification of eNOS signal, only arterial segments ≥200 µm in length were counted to eliminate the effect of varying angles on fluorescence intensity [25].

Levels of α-smooth muscle actin (mouse α-SMA, Santa Cruz Biotechnology, Inc.), neutrophil myeloperoxidase (rabbit anti-MPO, Abcam Inc), macrophage-specific glycoprotein F4/80 (rabbit anti-F4/80, Abcam Inc), vascular endothelial growth factor receptor-2 (VEGFR2, rabbit, Cell Signaling, Whitby, ON, Canada), and CD31 (goat anti-CD31, R&D Systems Inc.) were measured in kidney corticomedullary sections by immunohistochemistry. Interstitial peritubular capillary density was estimated with CD31 staining as the percent positive area per field of view. All primary antibodies were visualized with ImmPRESS Polymer Detection Kits (Vector Laboratories, Inc., Newark, CA, U.S.A.). All images were acquired with the Zeiss Imager A1 microscope (Carl Zeiss, Oberkochen, Germany) equipped with the Olympus camera DP73 (Olympus Canada, Richmond Hill, ON, Canada). Images were analyzed by ImageJ software (NIH, Bethesda, MD, U.S.A.).

### *In situ* hybridization (ISH)

miR-486-5p, random sequence (negative control) miRNA, and positive control probes were prepared by Advanced Cell Diagnostics (Newark, CA, U.S.A.). ISH was performed on paraffin-embedded kidney sections using the miRNAscope™ HD (RED) Assay according to the manufacturer’s protocol (Advanced Cell Diagnostics).

### Immunoblots

Lysates from whole kidney or human umbilical vein endothelial cells (HUVECs) were resolved by SDS-polyacrylamide gel electrophoresis, transferred to nitrocellulose membranes, blocked in 5% milk for 1 h, and incubated for 16 h at 4°C with primary antibodies against PTEN, eNOS, VEGFR2 (all 1:1000, Cell Signaling), VEGFA (1:1000, Santa Cruz Biotechnology, Inc.), and ICAM-1 (1:1000, R&D Systems (rat), 1:1000, Cell Signaling; human). Loading controls β-actin and glyceraldehyde 3-phosphate dehydrogenase (GAPDH) (both 1:4000, Cell Signaling) were incubated for 1 h at room temperature. Washed membranes were incubated with horseradish peroxidase-conjugated secondary antibodies anti-rabbit (1:5000, Abcam), anti-mouse or anti-goat (1:2000 or 1:5000, Jackson ImmunoResearch, West Grove, PA, U.S.A.) for 1 h at room temperature, and visualized by chemiluminescence. Densitometry was performed using ImageJ software (NIH) Bethesda, MD, U.S.A.

### Cell culture and hypoxia/reoxygenation (H/R)

HUVECs were obtained from American Type Culture Collection (ATCC, via Cedarlane Corp., Burlington, ON, Canada) and cultured at 37°C in 5% CO_2_ in EBM2 medium supplemented with microvascular growth factors and 2% fetal bovine serum (Lonza, Basel, Switzerland, catalog # CC-3156). Cells were transfected with 1 nM miR-486-5p mimic or scramble miRNA in Lipofectamine RNA-iMax (all from Thermo Fisher Scientific), and subjected to H/R as described [24].

### Blood pressure

Systolic blood pressure (SBP) was measured via tail-cuff plethysmography (CODA®, Kent Scientific Corp, Torrington, CT, U.S.A.). Eight weeks after surgery, average SBP was calculated from sessions comprising 15 cycles each to achieve at least 10 accepted readings. Rats underwent 3–4 days of training prior to measurements.

### Wire myography

Rat mesenteries were dissected and placed in ice-cold physiological saline solution (PSS). Segments (1.6–2.0 mm) of second-order branches (vessel diameter 300–550 µm) were mounted onto a Multi-Wire Myograph system (DMT, Ann Arbor, MI, U.S.A.) and equilibrated in PSS with 95% O_2_ and 5% CO_2_ at 37°C. Initial vessel viability was confirmed by contractile response to 60 mM potassium chloride (KCl), and vascular function assessment was performed by addition of acetylcholine (Ach, 10 µM) to vessels pre-constricted with phenylephrine (5 µM). Only vessels with viable endothelium (minimum 45% vasodilatory response of phenylephrine pre-constricted vessels to Ach) were included in analyses. Endothelium-dependent and -independent vasorelaxation was evaluated in response to Ach or sodium nitroprusside (SNP), respectively (1 nM to 10 µM). Data are presented as percentage of maximal constriction.

### Statistical Analyses

Results are expressed as mean ± SEM, and statistical comparisons were conducted using one- or two-way analysis of variance (ANOVA) with Tukey post-test as appropriate. Statistical analyses were performed with GraphPad Prism 9.4 (GraphPad Software, Inc., San Diego, CA, U.S.A.). Statistical significance was set at *P*<0.05.

## Results

### miR-486-5p localizes to the kidney and protects against ischemic injury

Preliminary experiments with the bilateral IR model in rat demonstrated peak elevation of plasma Cr after 24 h, with partial recovery by 72 h and return to baseline Cr by 4 weeks. A dose of 0.5 mg/kg of miR-486-5p was selected from preliminary experiments (*n*=3) showing efficacy.

**Figure 2:**
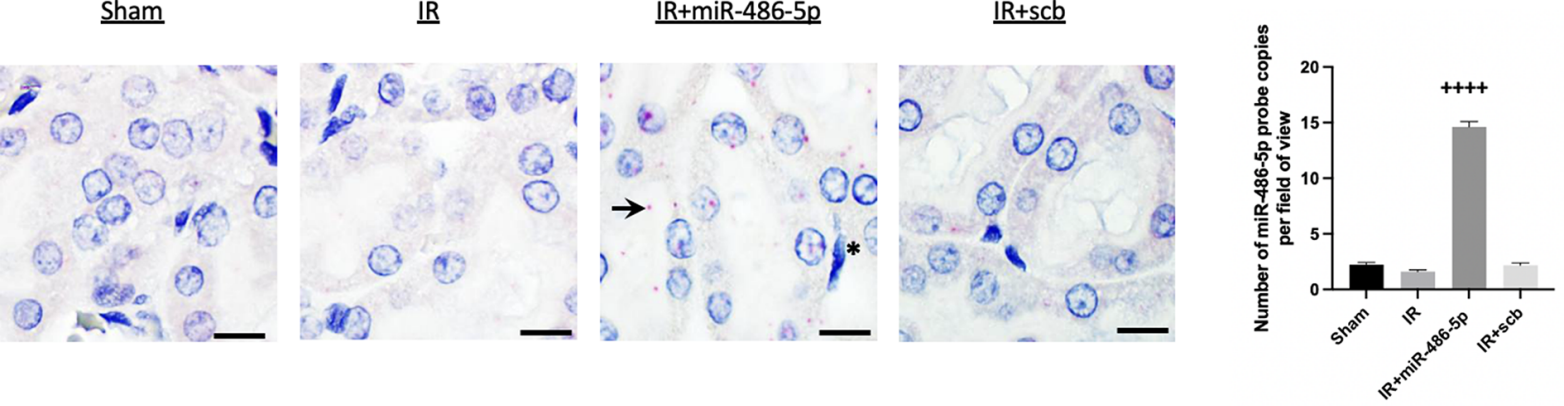
*In situ* hybridization for miR-486-5-p in rat kidney cortex Localization of miR-486-5p in the rat kidney cortex 24 h post-IR, with injection of miR-486-5p (0.5 mg/kg, IR+miR-486-5p) or scramble miRNA (0.5 mg/kg, IR+scb) at the time of reperfusion. At 24 h, enhanced miR-486-5p signal (pink-red areas) was observed in rats injected with miR-486-5p, localized to proximal tubular cells (arrow) and endothelial cells (asterisk). Background nuclear staining with hematoxylin. Scale bar = 10 µm, magnification ×1000. The graph shows the quantification of miR-486-5p probe copies/field of view (++++*P*<0.0001 vs all groups)

At 24 h after kidney IR injury, miR-486-5p levels were increased in the kidneys of rats administered miR-486-5p by real-time PCR (Supplementary Figure S1). miR-486-5p levels were also significantly increased in the liver and spleen of miR-486-5p-treated rats, but not in the heart. By *in situ* hybridization, rats treated with miR-486-5p demonstrated its localization within proximal tubular cells and interstitial capillary endothelial cells (Figure 2). Probe signal intensity was highest in rats that received miR-486-5p (Figure 2), and was absent in the negative control using a scramble probe. *In situ* hybridization did not detect miR-486-5p within macrophages from kidneys of rats with IR.

**Figure 3:**
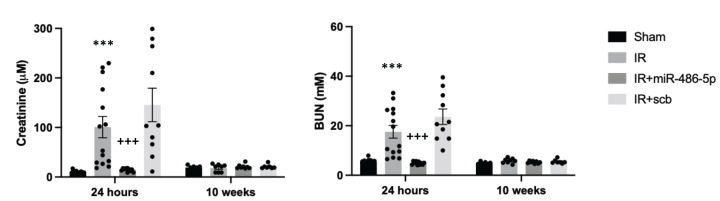
miR-486-5p protects against ischemic kidney injury in rat Graphs depict plasma creatinine (Cr, left) and blood urea nitrogen (BUN, right) at 24 h and 10 weeks after kidney IR injury, with administration of miR-486-5p or scramble (scb) miRNA by tail vein injection at the start of reperfusion. Plasma Cr and BUN are reported in standard SI units. To convert plasma Cr from µM to mg/dL, multiply by 0.0113, and to convert mM BUN to mg/dL, multiply by 2.8. ***P<0.001 vs sham, IR+miR-486-5p; +++*P*<0.001 vs IR+scb. Number of rats: *n* = 10–14 rats per group (24 h) and *n* = 6–8 rats per group (10 weeks).

**Figure 4:**
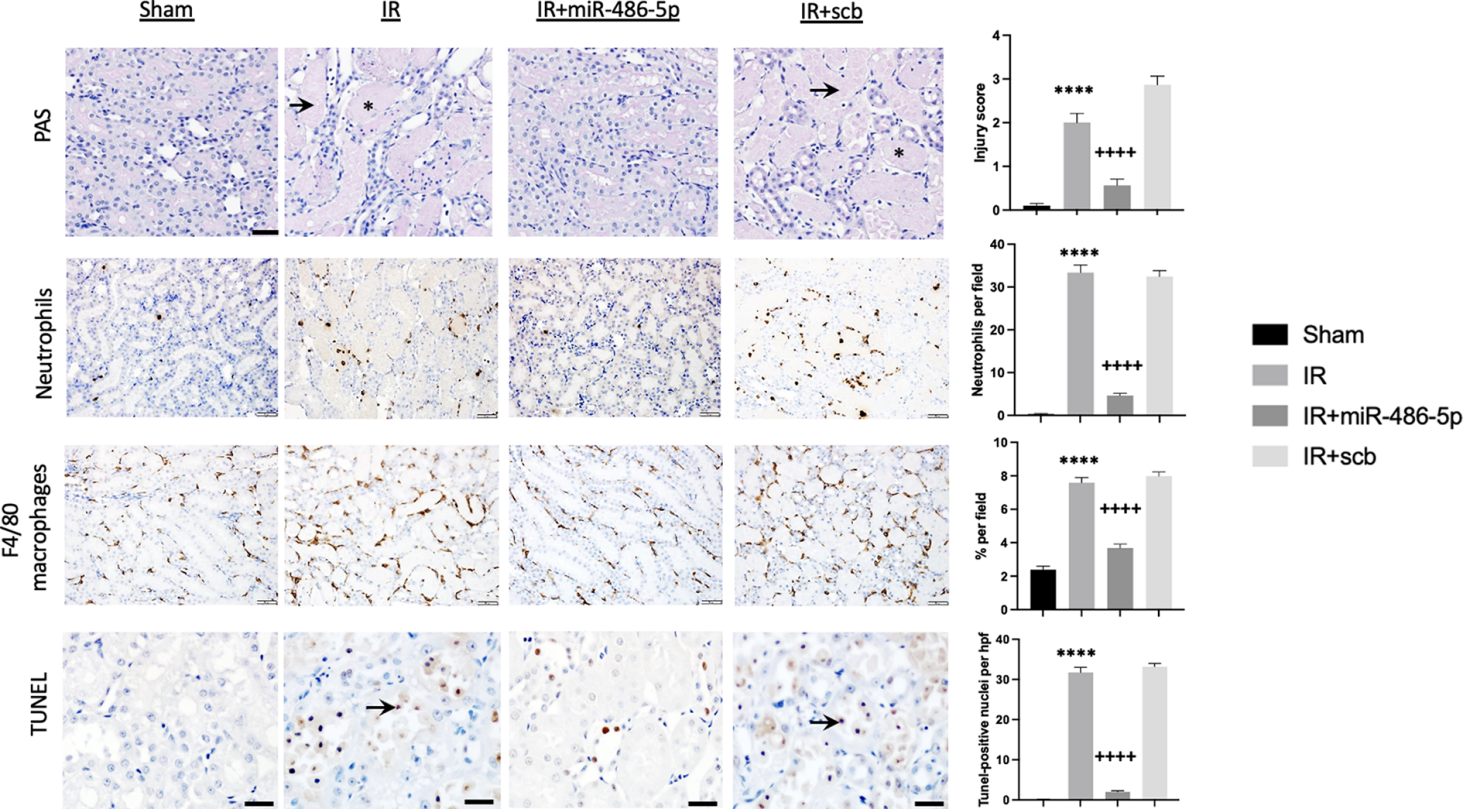
Effect of miR-486-5p on kidney injury scores, inflammatory cell infiltration and apoptosis 24 h after IR injury Groups include sham rats, rats with kidney IR injury alone (IR), or rats with kidney IR injury and treated with either miR-486-5p mimic (IR+miR-486-5p) or scramble miRNA (IR+scb) at the start of reperfusion. Representative images from sections of outer medulla. Periodic acid schiff (PAS) staining depicts acute tubular injury in IR and IR+scb rat groups (asterix: tubular cast; arrow: tubular dilatation, nuclear loss) (scale bar = 50 µm, magnification ×200). Representative images of kidney neutrophil and macrophage infiltration (scale bar = 50 µm, magnification ×200). Representative images of terminal deoxynucleotidyl transferase-mediated dUTP nick-end label (TUNEL) staining (scale bar = 20 µm, magnification ×400), with arrows indicating stained nuclei. Graphs on the right depict semiquantitative analyses. ****P<0.0001 vs sham, IR+miR-486-5p; ++++*P*<0.0001 vs IR+scb; *n* = 7 rats per group.

miR-486-5p potently protected against kidney ischemic injury, attenuating the ischemic injury-induced rise in plasma Cr and BUN levels (Figure 3). Laser doppler flowmetry demonstrated that miR-486-5p preserved cortical blood flow and enhanced medullary blood flow (Supplementary Figure S2). At 24 h, kidneys from miR-486-5p-treated rats showed decreased tubular injury, decreased infiltration of neutrophils and macrophages, and diminished apoptosis (Figure 4).

### Effect of miR-486-5p on endothelial protein levels

**Figure 5:**
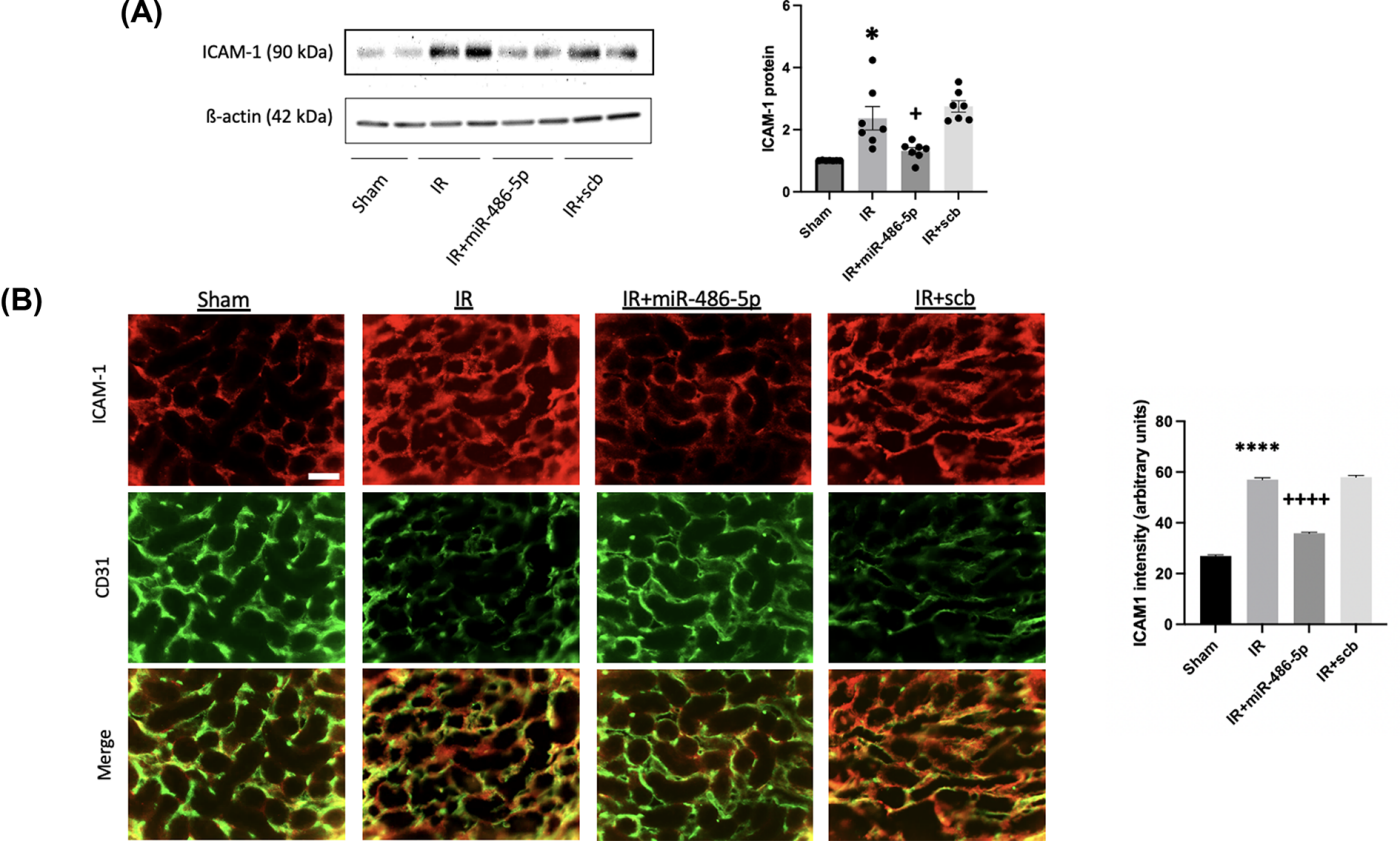
Effect of miR-486-5p on kidney protein levels of ICAM-1 24 h after IR injury (**A**) Graph shows kidney protein expression of ICAM-1 with representative immunoblot. Protein expression was normalized to β-actin. (**B**) Representative immunofluorescence images of ICAM-1 (red), CD31 (green), and co-localization (scale bar = 50 µm, magnification ×400) with semiquantative analysis for ICAM-1 signal ****P<0.0001, *P<0.05 vs sham, IR+miR-486-5p; ++++*P*<0.0001, +*P*<0.05 vs IR+scb miRNA. *n* = 7 rats per group.

Since miR-486-5p was detected within endothelial cells 24 h after injection and preserved regional kidney blood flow and kidney function, we performed immunoblots and histological analyses for proteins involves in vascular function and endothelial activation [3,24,26]. ICAM-1 was detected in capillary endothelium, with significantly increased levels after kidney IR injury by both immunoblot and immunofluorescence (Figure 5). This effect was blocked by miR-486-5p (Figure 5). We also transfected HUVECs with miR-486-5p and subjected them to H/R; this revealed significant attenuation of the H/R-induced up-regulation of ICAM-1 (Supplementary Figure S3).

**Figure 6:**
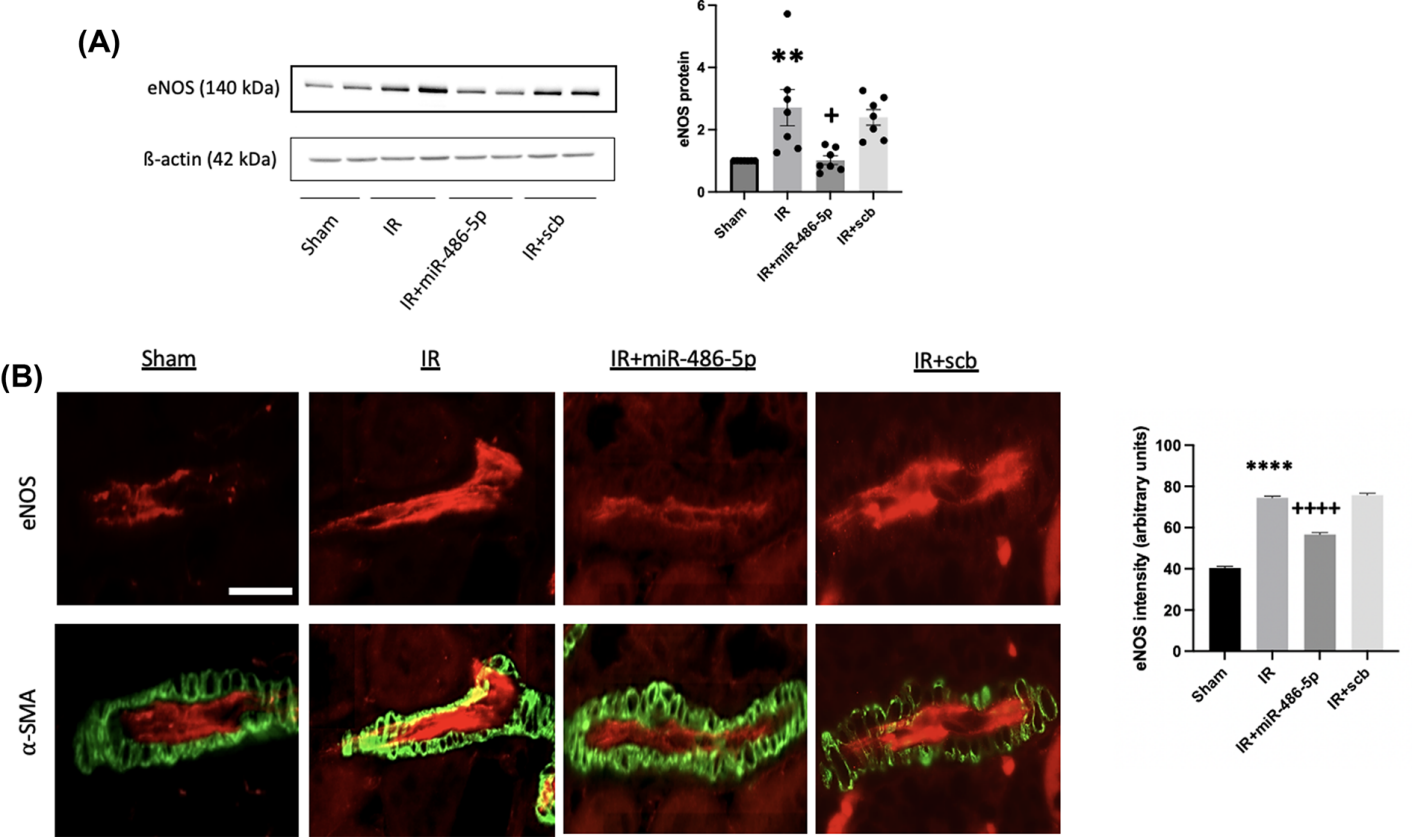
Effect of miR-486-5p on kidney protein levels of eNOS 24 h after IR injury (**A**) Graph shows kidney protein expression of eNOS with representative immunoblot. Protein expression was normalized to β-actin. (**B**) Representative immunofluorescence images of eNOS (red) and α-smooth muscle actin (α-SMA; green) in renal terminal arterioles (scale bar = 30 µm, magnification ×400) with semiquantitative analysis for eNOS signal. ****P<0.0001, **P<0.01 vs sham, IR+miR-486-5p; ++++*P*<0.0001, +*P*<0.05 vs IR+scb miRNA. *n* = 7 rats per group.

**Figure 7:**
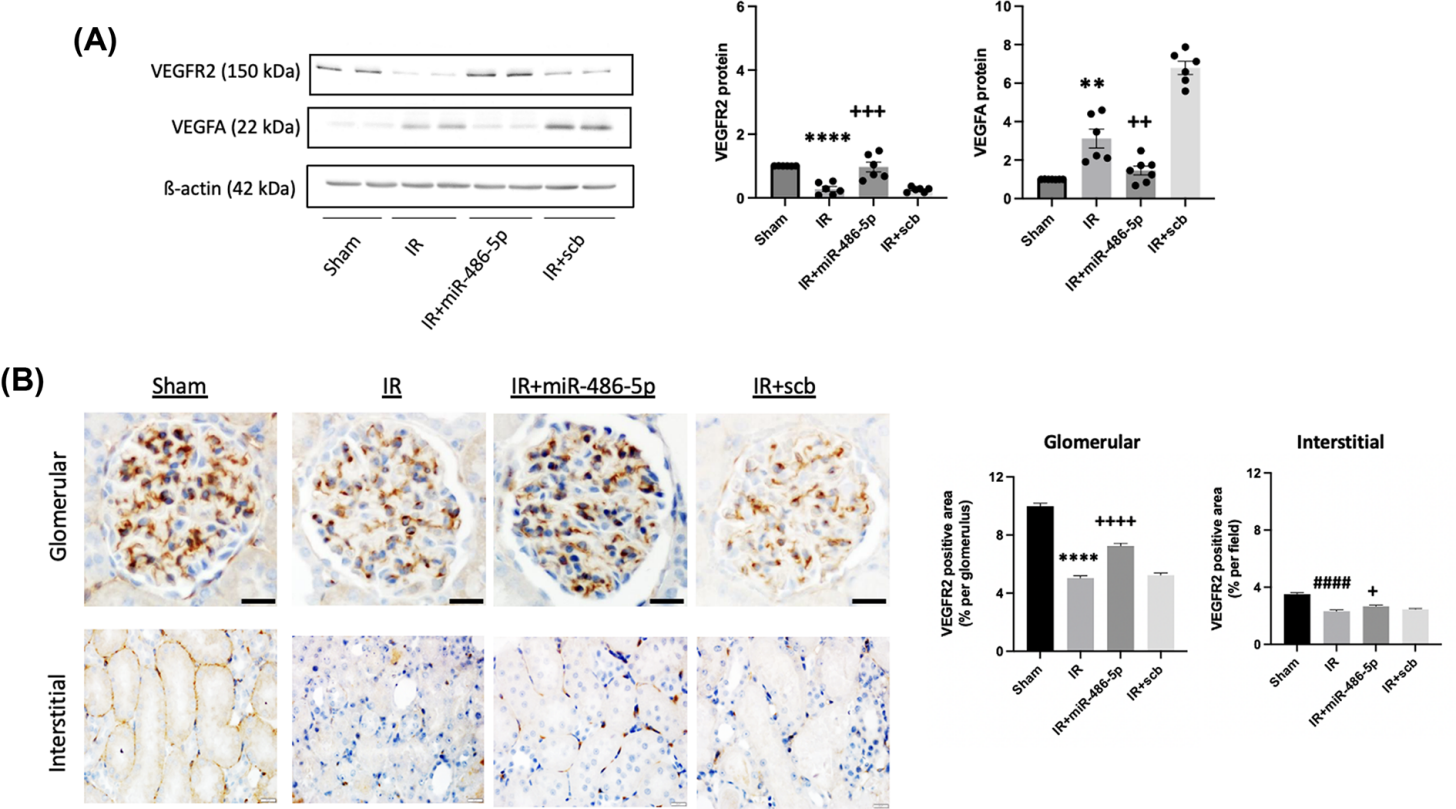
Effect of miR-486-5p on kidney protein levels of vascular endothelial growth factor receptor-2 (VEGFR2) and vascular endothelial growth factor-A (VEGFA) 24 h after IR injury (**A**) Graphs show kidney protein expression of VEGFR2 and VEGFA, with representative immunoblots. Protein expression was normalized to β-actin. (**B**) Representative images of VEGFR2 staining within glomeruli (top, scale bar = 20 µm, magnification ×400) and interstitium (bottom, scale bar = 20 µm, magnification ×400) by immunohistochemistry with semiquantitative analysis. ****P<0.0001, **P<0.01 vs sham, IR+miR-486-5p; ++++*P*<0.0001, +++*P*<0.001, ++*P*<0.01 vs IR+scb miRNA; +*P*<0.05 vs IR; ####*P*<0.0001 vs sham. *n* = 7 rats per group.

Kidney IR injury up-regulated eNOS protein levels, observed within endothelial cells of terminal arterioles, and this was inhibited by miR-486-5p on immunoblot and immunofluorescence (Figure 6). IR injury also increased whole kidney VEGFA by immunoblot analysis, and this was inhibited by miR-486-5p (Figure 7), although immunohistochemistry did not permit precise localization on either paraffin-embedded or frozen sections (not shown). To determine if the effects of miR-486-5p on eNOS and VEGFA protein levels were specific to kidney, we conducted immunoblot analysis on liver tissue isolated from the rat groups. The results show that kidney IR had no significant effect on liver expression of eNOS or VEGFA, and miR-486-5p injection did not alter levels (Supplementary Figure S4).

Decreased VEGFR2 levels were observed after kidney IR injury by immunoblot. Immunohistochemistry demonstrated staining localized within glomeruli and interstitial endothelial cells and showed that miR-486-5p attenuated this IR-induced decrease (Figure 7).

**Figure 8:**
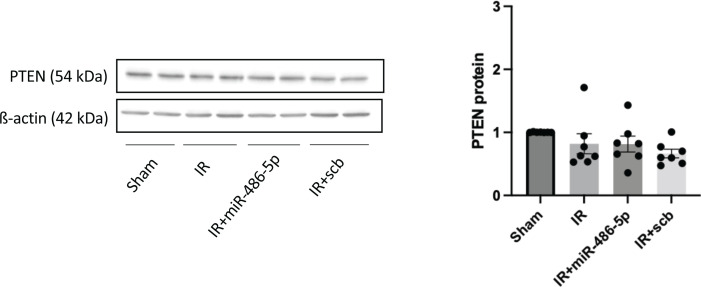
Effect of miR-486-5p on kidney protein levels of phosphatase and tensin homolog (PTEN) 24 h after IR injury in rats Graph shows kidney protein expression of PTEN with representative immunoblots. Protein expression was normalized to β-actin. *n* = 7 rats per group.

In our previous studies involving mice with kidney IR injury, administration of miR-486-5p was associated with decreased PTEN protein levels [16,17]. In rat, however, miR-486-5p had no effect on kidney PTEN protein levels at 24 h (Figure 8).

### Effect of early administration of miR-486-5p on peritubular capillary density and endothelial function at 10 weeks

**Figure 9:**
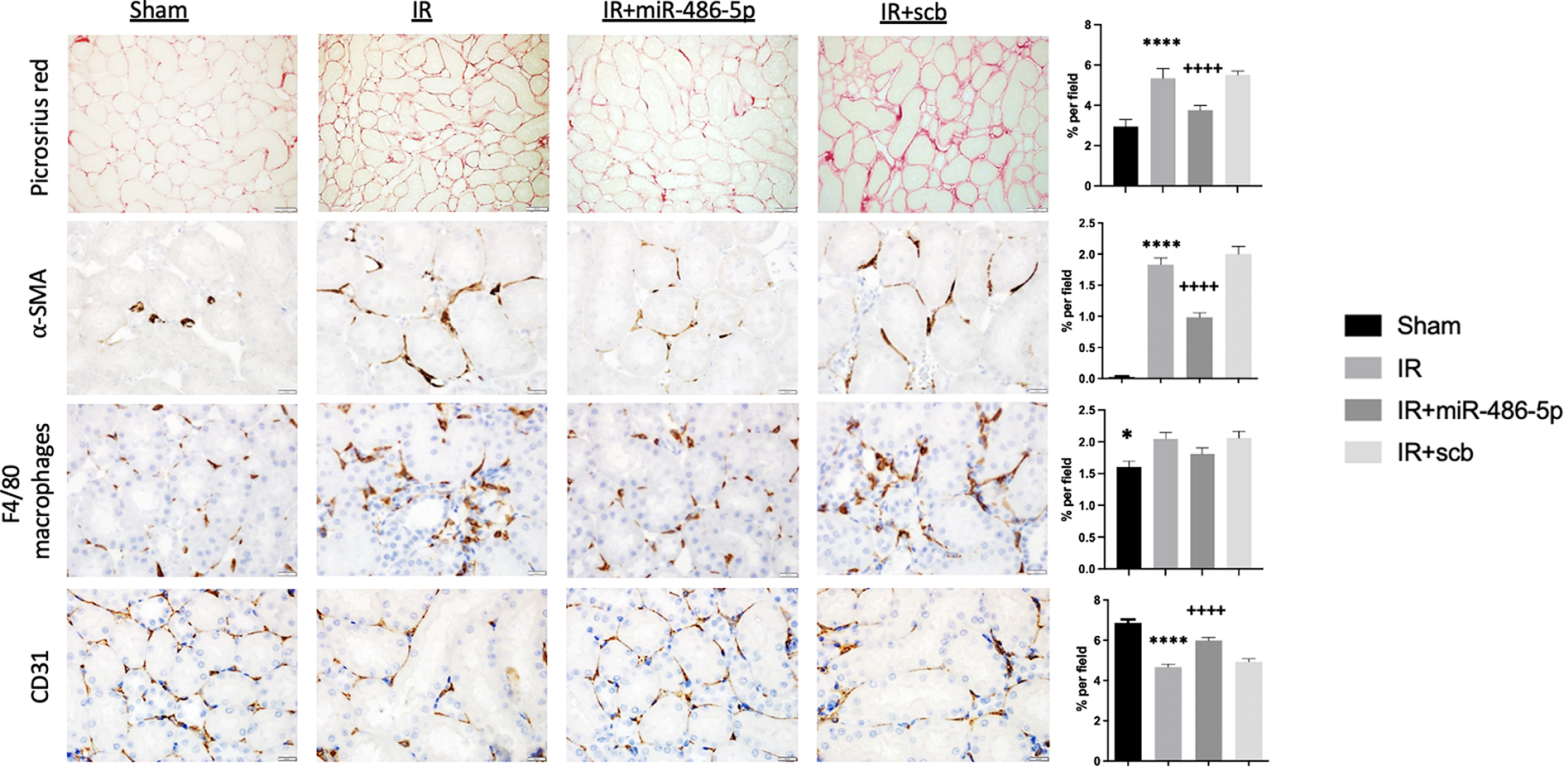
Effect of early miR-486-5p administration on kidney fibrosis and peritubular capillary density 10 weeks after kidney IR injury Semiquantitative analysis and representative images of picrosirius red staining for interstitial collagen deposition (scale bar = 50 µm, magnification ×200), smooth muscle ⍺-actin (⍺-SMA, scale bar = 20 µm, magnification ×400), kidney macrophage infiltration (F4/80, scale bar = 20 µm, magnification ×400), and CD31^+^ peritubular capillary density (scale bar = 20 µm, magnification ×400) from kidneys of sham rats, rats with kidney IR injury (IR) alone, or rats with kidney IR injury administered either miR-486-5p (IR+miR-486-5p) or scramble miRNA (IR+scb) at the start of reperfusion. ****P<0.0001 vs sham, IR+miR-486-5p, *P<0.05 vs IR; ++++*P*<0.0001 vs IR+scb. *N* = 6–8 rats per group.

Since miR-486-5p inhibited ischemia-stimulated kidney eNOS and VEGF protein at 24 h, rats were followed for 10 weeks to determine the impact on development of capillary rarefaction and interstitial fibrosis [13,27]. At 10 weeks, rats with IR injury alone recovered kidney function with normalization of plasma Cr and BUN (Figure 2) and without significant increase in albuminuria (Supplementary Figure S5). Kidneys from these rats had increased collagen fiber content by picrosirius red staining and decreased CD31^+^ peritubular capillary density (Figure 9). Post-ischemic kidneys showed increased staining for the pro-fibrotic myofibroblast marker α-SMA, and F4/80^+^ macrophages (Figure 9). By contrast, kidneys from rats treated with miR-486-5p had preserved peritubular capillary density and decreased staining for interstitial collagen fibers, α-SMA and F4/80 macrophages.

**Figure 10:**
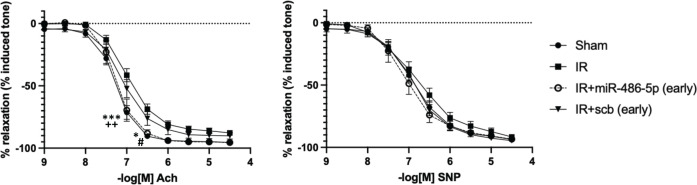
Effect of early administration of miR-486-5p on mesenteric artery reactivity 10 weeks after kidney IR injury Left: Acetylcholine (Ach), Right: Sodium nitroprusside (SNP); ***P<0.001, *P<0.05, sham vs IR, IR+scramble (scb); ++*P*<0.01, IR+miR-486-5p vs IR, IR+scb, #*P*<0.05, IR+miR-486-5p vs IR; *n* = 9–14 rats per group for sham and IR groups, and *n* = 6–7 rats per group for IR+miR-486-5p and IR+scb groups.

Recovery from AKI is associated with impaired kidney hemodynamics [28], hypertension [28,29], and peripheral vascular dysfunction [29]. At 10 weeks, all groups of rats had similar kidney cortical and medullary blood flows, and systolic blood pressure (SBP) (Supplementary Figure S6). However, mesenteric arteries from rats with IR alone (or treated with scb miRNA) showed impaired endothelium-dependent vasorelaxation, which was prevented by early administration of miR-486-5p (Figure 10). There were no significant differences in the endothelium-independent vasodilatory responses to SNP among the groups.

### Late administration of miR-486-5p does not affect AKI to CKD transition

We next determined if late administration of miR-486-5p might affect vascular function or transition to CKD. Rats were administered a first dose of miR-486-5p (0.5 mg/kg i.v.) at 96 h after IR, and a second dose at 3 weeks. At 24 h after the first dose, RT-qPCR revealed increased kidney levels of miR-486-5p (Supplementary Figure S7). At that time point (120 h after kidney IR), kidney eNOS protein expression remained significantly increased in rats with IR alone, but not in rats with IR injury treated with miR-486-5p (Supplementary Figure S7).

**Figure 11:**
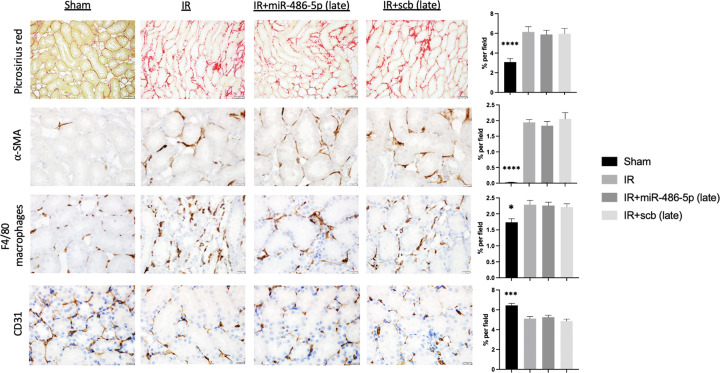
Effect of delayed miR-486-5p administration on kidney fibrosis and peritubular capillary density 10 weeks after IR injury Semiquantitative analysis and representative images of picrosirius red staining for interstitial collagen deposition (scale bar = 50 µm, magnification ×200), smooth muscle ⍺-actin (⍺-SMA, scale bar = 20 µm, magnification ×400), kidney macrophage infiltration (F4/80, scale bar = 20 µm, magnification ×400), and CD31^+^ peritubular capillary density (CD31^+^, scale bar = 20 µm, magnification ×400) from kidneys of sham rats, rats with kidney IR injury alone (IR), or rats with kidney IR injury administered either miR-486-5p (IR+miR-486-5p) or scramble miRNA (IR+scb) at 96 h and 3 weeks after kidney IR injury. ****P<0.0001, ***P<0.001, *P<0.05, sham vs all groups, *n* = 6 rats per group.

**Figure 12:**
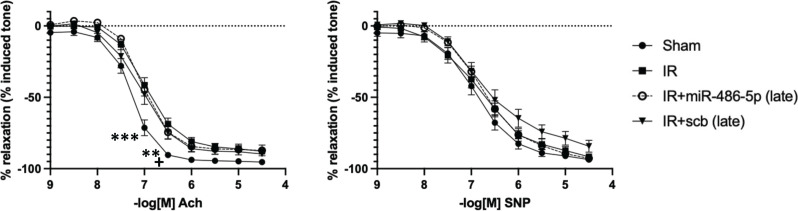
Effect of late administration of miR-486-5p on mesenteric artery reactivity 10 weeks after kidney IR injury Left: Acetylcholine (Ach), Right: Sodium nitroprusside (SNP); ***P<0.001, sham vs all groups, **P<0.01, sham vs IR, IR+miR-486-5p, +*P*<0.05, sham vs IR+ scramble miRNA (scb); *n* = 9–14 rats per group for sham and IR groups and *n* = 6 rats per group for IR+miR-486-5p and IR+scb groups.

In all rats subjected to kidney IR injury, plasma Cr and BUN were significantly increased 24 h after IR, with return towards baseline by 96 h, and complete recovery by 3 weeks, sustained to 10 weeks (Supplementary Figure S8). Late and repeated miR-486-5p dosing did not prevent development of kidney fibrosis or preserve capillary density at 10 weeks (Figure 11). Similarly, unlike the protective effect of early miR-486-5p treatment, delayed administration of miR-486-5p did not preserve Ach-mediated vasodilation in mesenteric arteries at 10 weeks (Figure 12).

## Discussion

Treatment options for AKI are limited [30]. We previously showed that direct administration of miR-486-5p protects against kidney IR injury in mice, associated with down-regulation of PTEN expression [17]. Although pre-clinical studies support the pro-angiogenic potential of miR-486-5p [19,20], an anti-angiogenic effect has been reported in cultured endothelial cells [21]. Furthermore, persistent PTEN loss following kidney injury may promote dysfunctional tubule regeneration and fibrosis [18]. We hypothesized that early administration of miR-486-5p would prevent ischemic AKI and the transition to CKD, with potential for delayed miR-486-5p administration to exert a negative impact on the vasculature. Our results indicate that miR-486-5p protects against ischemic AKI in rat, associated with inhibition of ICAM-1 up-regulation and decreased inflammatory cell infiltration. Early administration of miR-486-5p prevented peritubular capillary loss, interstitial fibrosis, and systemic endothelial dysfunction after 10 weeks, whereas delayed administration of miR-486-5p after peak kidney injury had no impact on long-term kidney or systemic vascular outcomes.

The mechanism by which miR-486-5p prevents ischemic AKI remains unclear but may involve multiple gene targets. In the present studies, *in situ* hybridization localized miR-486-5p within rat proximal tubular cells and interstitial capillary endothelial cells, suggesting a direct effect of miR-486-5p on these cells. Although miR-486 levels are increased within mouse kidney macrophages after unilateral ureteral obstruction [31], we were unable to detect miR-486-5p in kidney macrophages in rats with ischemic injury alone or rats treated with scramble miR, suggesting that endogenous levels are below detectability by this method. Relatively few macrophages were present within kidneys from mice treated with miR-486-5p, precluding quantification by *in situ* hybridization. Taken together, our data suggest that macrophage miR-486-5p is unlikely to contribute to the protective effect.

We showed that miR-486-5p inhibited IR-induced up-regulation of ICAM-1, eNOS, and VEGFA while preserving VEGFR2 expression. Since these are not predicted targets of miR-486-5p, the regulatory mechanism remains unknown. We previously identified differentially expressed genes involved in apoptosis and TNF-α signaling in proximal tubular cells from mice with kidney IR injury treated with miR-486-5p [17]. In pre-clinical models of kidney [17] and cardiac [32,33] IR injury, miR-486-5p decreases PTEN protein expression and inhibits apoptosis. miR-486-5p also transiently down-regulated kidney PTEN mRNA at 4 h after kidney IR injury in mice [17], yet the present study found no effect of miR-486-5p on PTEN protein expression at 24 h after kidney IR injury in rat. It is plausible that early, transient down-regulation of miR-486-5p target gene expression contributes to its protective effect while minimizing potential long-term negative consequences. In this regard, sustained PTEN knockdown in kidney IR injury confers a pro-fibrotic phenotype [18], while pharmacological inhibition of PTEN has been shown by Zhou et al. to exacerbate ischemic kidney injury in mice [34].

In kidney IR injury, outer medullary hypoperfusion exacerbates tubular epithelial cell injury and contributes to permanent peritubular capillary loss [35]. Collett et al. demonstrated that infusion of conditioned medium from endothelial-colony forming cells (ECFCs) protected against rat kidney IR injury and early loss of medullary blood flow, and inhibited IR-induced ICAM-1 expression [10]. Interestingly, we previously showed that cord blood ECFC-derived conditioned medium contains small extracellular vesicles (exosomes) that inhibit ICAM-1 up-regulation in HUVECs subjected to H/R injury [24], and confer a protective effect against kidney IR injury in mice via exosomal transfer of miR-486-5p [16]. In the present studies, miR-486-5p preserved renal blood flow and attenuated endothelial injury as evidenced by inhibition of IR-induced ICAM-1 up-regulation along with decreased inflammatory cell infiltration. Although ICAM-1 is not a predicted direct miR-486-5p target, miR-486-5p inhibited its up-regulation *in vivo* and *in vitro* in transiently transfected HUVECs subjected to H/R injury. These results strongly suggest that miR-486-5p preserves endothelial function by targeting pathways involved in endothelial cell activation and leukocyte adhesion/migration. Indeed, targeting of this pathway by miR-486-5p may represent an important mechanism for its protective effect in ischemic AKI.

Although eNOS is also not a predicted direct target of miR-486-5p, its administration early or late after IR injury decreased total kidney eNOS expression compared with rats with IR alone. Other studies have reported IR-induced up-regulated eNOS protein levels [36,37], presumably as a protective mechanism to preserve kidney perfusion. Nitric oxide (NO) is an important regulator of kidney microvascular blood flow [38] with evidence for beneficial effects [5,39–41] but also potential for worsening [42] in kidney IR injury. In this regard, both non-selective NOS or eNOS inhibition decrease apoptosis after kidney IR injury, and eNOS activity has been linked to inducible NOS (iNOS) overexpression and increased apoptosis [43,44]. IR-induced eNOS up-regulation may also contribute to eNOS uncoupling, a phenomenon that results in superoxide and free radical production and scavenging of NO to form peroxynitrite [45,46]. Further, in mice, eNOS overexpression increased liver IR injury, inducing protein nitration, transaminitis, and apoptosis [47]. Thus, it is plausible that inhibition of IR-induced eNOS expression by miR-486-5p might prevent injury related to reactive nitrogen species. On the other hand, our data indicate that miR-486-5p preserves endothelial function, and enhanced NO production as a response to IR may not be necessary. Further studies are required to determine the role of miR-486-5p-induced inhibition of eNOS expression in the protective response to kidney IR injury.

AKI is associated with long-term increased risk of systemic vascular disease and hypertension [48]. Post-ischemic rats developed mesenteric artery dysfunction at 10 weeks, while early administration of miR-486-5p preserved endothelium-dependent vasorelaxation. In mice, systemic vascular dysfunction with impaired endothelium-dependent vasorelaxation has been reported 4 weeks after kidney IR injury, with a role for activation of the endothelin system as mediator [29]. While the role of miR-486-5p in modulating the endothelin system after kidney IR injury is unknown, in human pulmonary artery smooth muscle cells miR-486-5p increased the expression and secretion of endothelin-1 [49]. However, the effect of miR-486-5p on biological pathways via its target genes depends on cell type or experimental model [50].

In this study, we showed that delayed administration of miR-486-5p at 96 h and 3 weeks after kidney IR injury did not prevent peritubular capillary rarefaction, development of tubulointerstitial fibrosis, or systemic endothelial dysfunction. In rats with kidney IR injury, Leonard et al. showed that VEGF administration preserved microvascular density when administered early post-IR, but lacked effect on vessel recovery when administered late (3 weeks) after IR [36]. miR-486-5p has an anti-fibrotic effect in pre-clinical models of pulmonary and cardiac fibrosis via targeting of Smad1/2 and inhibition of transforming growth factor (TGF)-β signaling [51,52]. The pathogenesis of tubulointerstitial fibrosis after kidney IR injury is likely complex beyond TGF-β signaling, involving permanent microvascular damage [13,27] and release of inflammatory mediators such as high mobility group box-1 (*HMGB1*) from injured or necrotic tubular cells [53]. In mice, early delivery of miR-486-5p targets genes involved in injury pathways in kidney IR, including TNF-α signaling and apoptosis [17]. In rat, we show that miR-486-5p inhibits endothelial activation and preserves endothelial function. Early administration of miR-486-5p after kidney IR injury is therefore critical to prevent permanent peritubular capillary loss and the transition to CKD, and indeed our data suggest this process is programmed to occur soon after reperfusion.

Our study has certain limitations. First, experiments were performed exclusively on male rats. In this regard, however, in previous studies, we showed that female mice are significantly less susceptible to kidney IR injury [54]. Second, considerable variability in extent of injury 24 h after reperfusion was observed in the rat model of bilateral IR, a feature that is widely recognized [55]. Third, we used a volatile anesthetic (isoflurane) in our rat kidney IR model, as such anesthetics are routinely administered in clinical practice, including cardiovascular surgeries that represent an important cause of kidney IR injury in humans. We recognize that volatile anesthetics have renoprotective effects [56], although we carefully controlled for this issue by providing all rats with the same anesthetic treatment. Fourth, SBP evaluation by non-invasive tail cuff plethysmography may not have been sensitive enough to detect mild but significant changes in blood pressure. Furthermore, laser doppler flowmetry is ideally suited for measuring relative blood flow changes, and not absolute flow.

In summary, miR-486-5p administered as a single dose at the start of reperfusion protects against ischemic AKI in rat, preserves early endothelial function, prevents peritubular capillary loss and tubulointerstitial fibrosis and preserves endothelium-dependent vasorelaxation. Further, despite inhibition of eNOS protein expression, delayed administration of miR-486-5p after peak injury has no long-term adverse or protective effects. These results suggest that miR-486-5p is a promising treatment for the prevention of ischemic AKI and associated long-term complications.

## Clinical perspectives

- IR acute kidney injury is associated with permanent reduction of peritubular capillary density, tubulointerstitial fibrosis, and transition to CKD. miR-486-5p protects against kidney IR injury in mouse, but its long-term effect on the vasculature and CKD development is unknown.
- In rats with kidney ischemia, miR-486-5p administered at the start of reperfusion inhibited ischemia-induced endothelial intercellular adhesion molecule-1 and preserved kidney function, despite inhibiting up-regulation of kidney eNOS. After 10 weeks, rats with early administration of miR-486-5p showed no evidence of CKD, or systemic endothelial dysfunction.
- The present study highlights the potent protective effects of early intervention with miR-486-5p on rat kidney ischemic injury and its long-term complications. The prevention of late capillary loss and vascular dysfunction by miR-486-5p support the critical importance of early gene responses in programing the transition from acute to chronic kidney disease.

## Supplementary Material

Supplementary Figures S1-S8

## Data Availability

All data are available by contacting the corresponding author.

## Abbreviations

AKI: acute kidney injury
Ach: Acetylcholine
CKD: chronic kidney disease
Cr: creatinine
ECFC: endothelial-colony forming cell
eNOS: endothelial nitric oxide synthase
*HMGB1*: high mobility group box-1
HUVEC: human umbilical vein endothelial cells
ICAM-1: intercellular adhesion molecule-1
iNOS: inducible NOS
IR: ischemia–reperfusion
NO: nitric oxide
PTEN: phosphatase and tensin homolog
SBP: systolic blood pressure
SMA: smooth muscle actin
SNP: sodium nitroprusside
TGF-β: transforming growth factor-β
3' UTR: 3' untranslated region
VEGF: vascular endothelial growth factor
VEGFR2: vascular endothelial growth factor receptor 2
