# Supplementary material for: miR-486-5p protects against rat ischemic kidney injury and prevents the transition to chronic kidney disease and vascular dysfunction
Source: Clin Sci (Lond). 2024 May 22;138(10):599–614. doi: 10.1042/CS20231752 (PMC11130553; doi:10.1042/CS20231752)
Supplement: Supplementary Figures S1-S8 [file CS-2023-1752_supp.pdf]

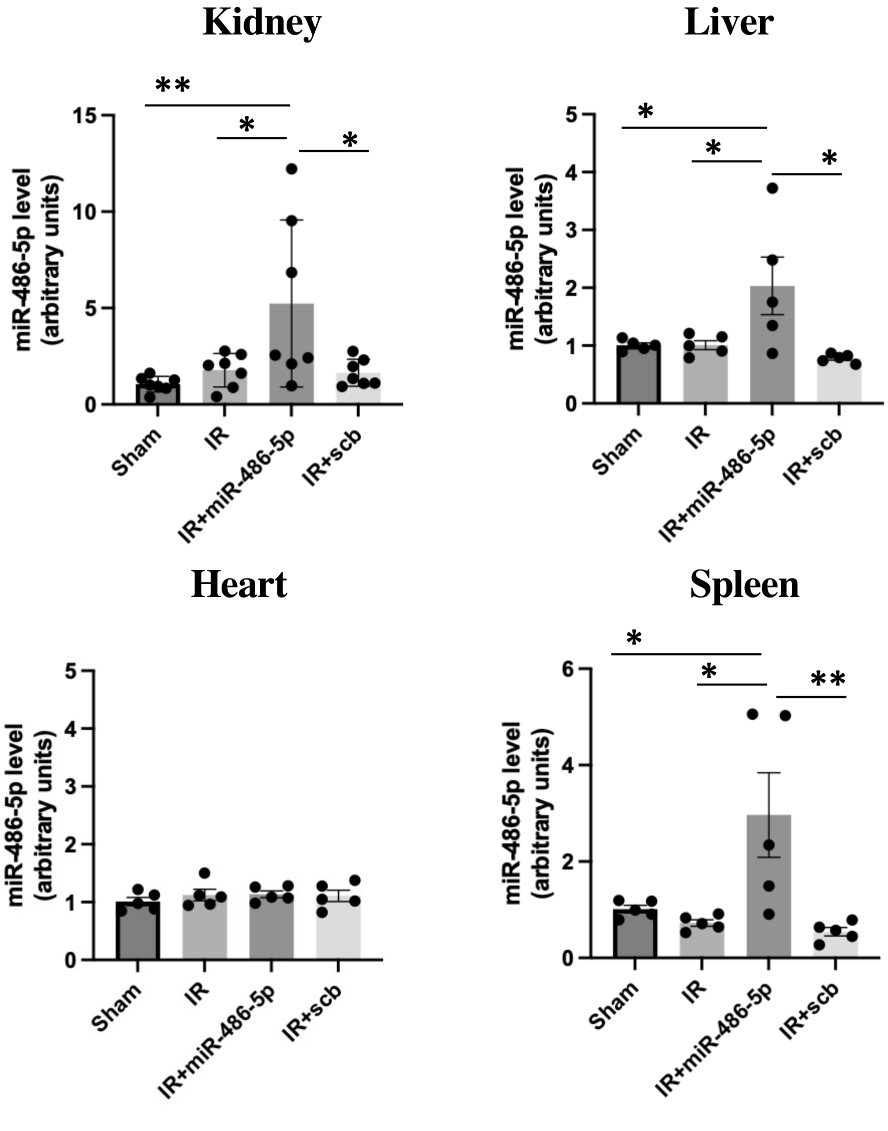

Figure S1. miR-486-5p levels in rat kidney, liver, heart and spleen at 24 h after kidney ischemia-reperfusion (IR) injury. Groups include sham-operated, kidney ischemia-reperfusion injury alone (IR), or kidney IR injury with administration of 0.5 mg/kg miR-486-5p (IR+miR-486-5p) or scramble miRNA (IR+scb) by tail vein injection at the start of reperfusion. miR-486-5p levels were normalized to endogenous U6 snRNA. **Kidney**: **$p<0.01$ for sham vs IR+miR-486-5p; *$p<0.05$ for IR and IR+scb vs IR+miR-486-5p; **Liver:** *$p<0.05$ for sham and IR+scb vs IR+miR-486-5p. **Spleen**: *$p<0.05$ for sham and IR vs IR+miR-486-5p, **$p<0.01$ for IR+scb vs IR+miR-486-5p. N=7 rats per group for kidney, and N=5 rats per group for liver, heart, and spleen.

Supplementary figure 1

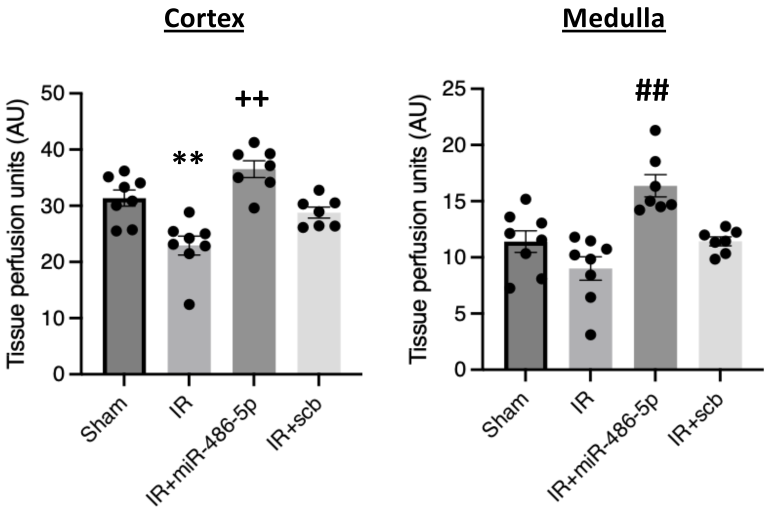

Figure S2. **Effect of miR-486-5p on regional kidney blood flow 24 h after ischemia-reperfusion (IR) injury in rat.** Cortical (left) and medullary (right) blood flows by laser doppler flowmetry 24 h after kidney IR injury. **p<0.01 vs sham, IR+miR-486-5p; ++p<0.01 vs IR+scb; ##p<0.01 IR+miR-486-5p vs all groups; n=7 rats per group.

Supplementary figure 2

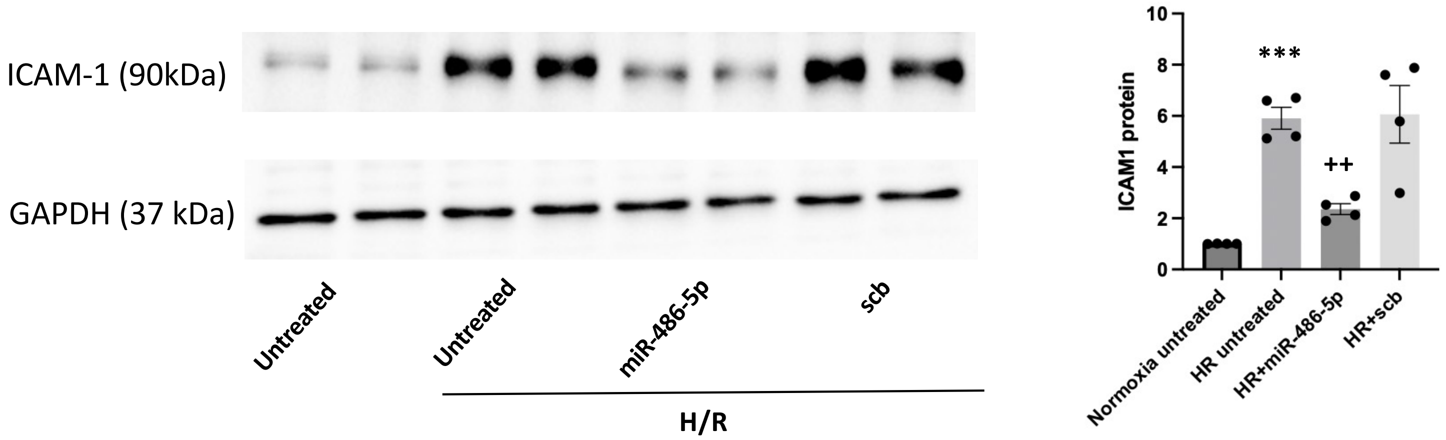

Figure S3. Effect of miR-486-5p on intercellular adhesion molecule-1 (ICAM-1) protein levels in HUVECs subjected to hypoxia/reoxygenation (H/R). ICAM-1 protein expression was evaluated by immunoblot and normalized to glyceraldehyde-3-phosphate dehydrogenase (GAPDH). Representative immunoblot is shown. Groups include untreated normoxic control HUVECs, and untreated, miR-486-5p- or scramble miRNA-transfected HUVECs exposed to H/R (H/R untreated, H/R+miR-486-5p, H/R+scb). ***p<0.001 H/R untreated vs normoxia untreated and H/R+miR-486-5p; ++p<0.01 H/R+miR-486-5p vs H/R+scb (n=4).

Supplementary figure 3

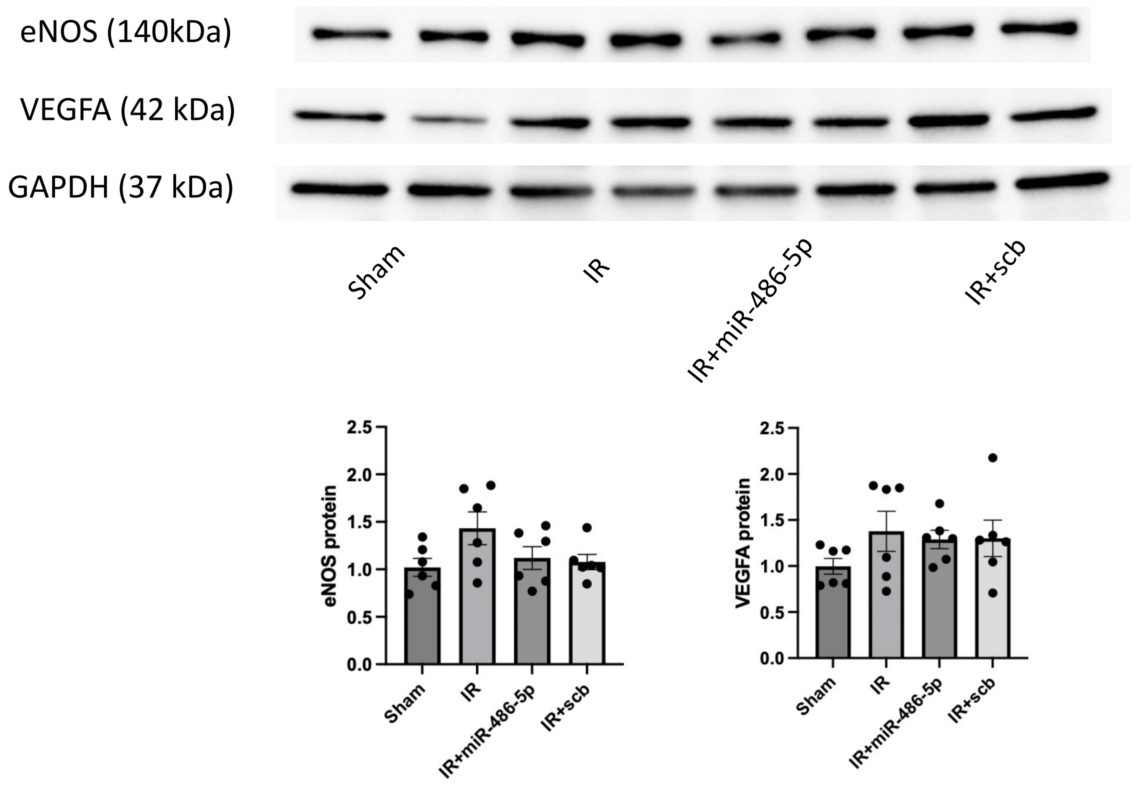

Figure S4: Effect of miR-486-5p on liver protein levels of endothelial nitric oxide synthase (eNOS) and vascular endothelial growth factor-A (VEGFA) 24 h after ischemia-reperfusion (IR) injury in rats. Graphs show liver protein expression of eNOS and VEGFA (dimer only) with representative immunoblots. Protein expression was normalized to GAPDH (n=6 rats per group)

Supplementary figure 4

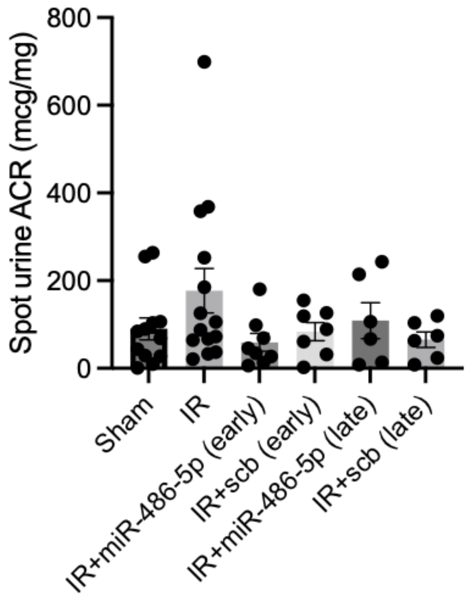

Figure S5. **Urine albumin to creatinine ratio (ACR) in rats 10 weeks after bilateral kidney ischemia-reperfusion (IR) injury, with early or late administration of miR-486-5p.** Groups include sham-operated, kidney IR injury alone (IR), kidney IR injury with early administration of miR-486-5p or scramble miRNA (scb) at the start of reperfusion, and kidney IR injury with late administration of miR-486-5p (or scb miRNA) at 96 h (dose #1) and 3 weeks (dose #2) after reperfusion. n=12-14 rats per group for sham and IR groups, and n=6-8 rats per group for IR+miR-486-5p and IR +scb groups

Supplementary figure 5

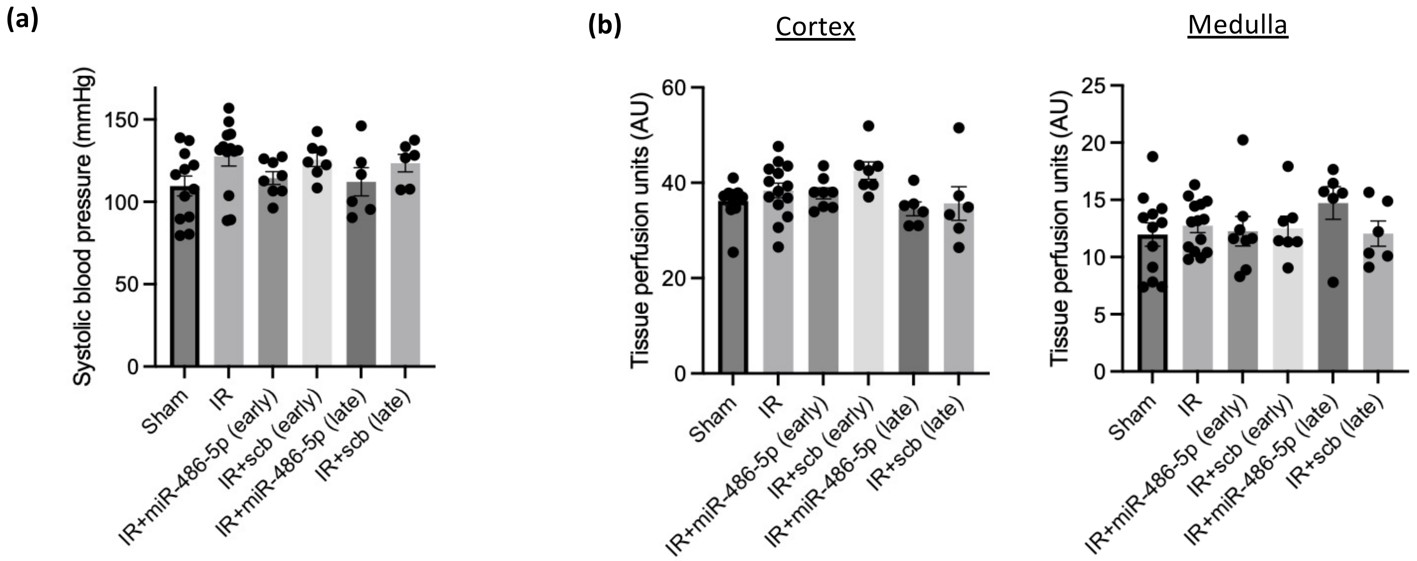

Figure S6. Blood pressure and regional kidney blood flow 8-10 weeks after kidney ischemia-reperfusion (IR) injury. (A) Systolic blood pressure 8 weeks after kidney IR injury. (B) Kidney cortical and medullary blood flows 10 weeks after kidney IR injury; n=12-14 rats per group for sham and IR groups, and n=6-8 rats per group for IR+miR-486-5p and IR+scramble (scb) groups.

Supplementary figure 6

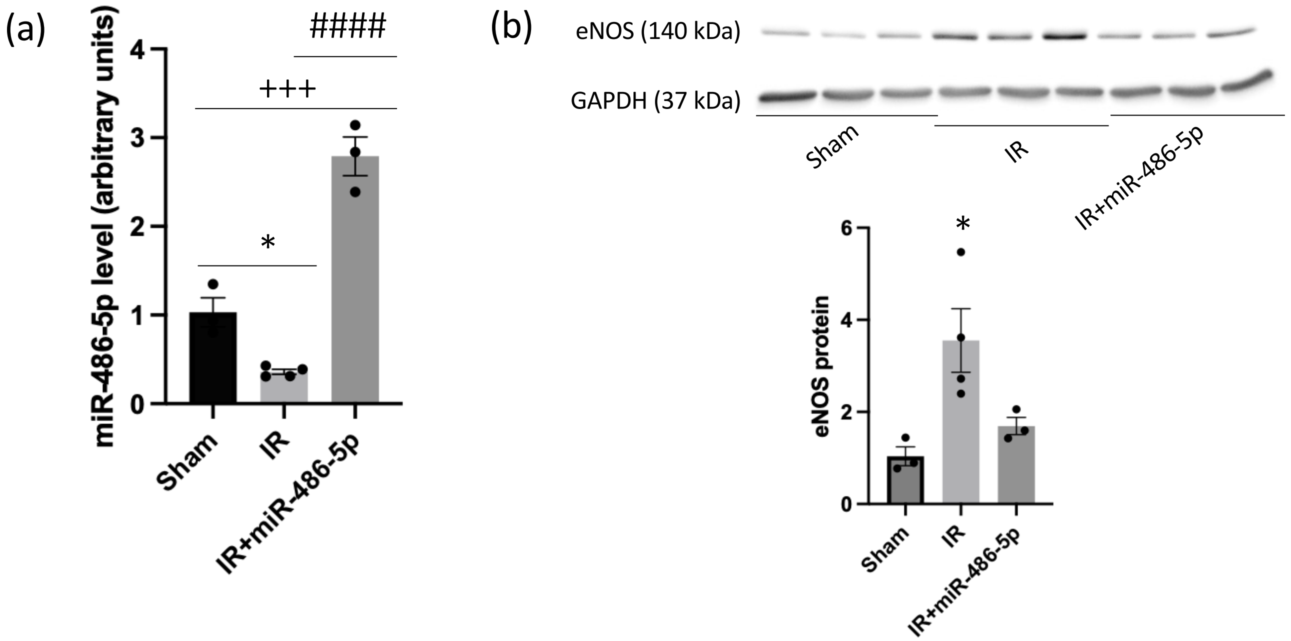

Figure S7. Kidney miR-486-5p levels (a) and endothelial nitric oxide synthase (eNOS) protein expression (b) 24 h after late administration of miR-486-5p. Rats were subjected to kidney ischemia-reperfusion (IR) injury, followed by administration of miR-486-5p 96 h after reperfusion and sacrifice 24 h later. Rat groups include sham-operated, kidney ischemia-reperfusion (IR) injury alone (IR), or kidney IR injury with administration of 0.5 mg/kg miR-486-5p (IR+miR-486-5p) (a) miR-486-5p levels were normalized to endogenous U6 snRNA. *p<0.05 (sham vs IR), +++p<0.001 (sham vs IR+miR-486-5p), ####p<0.0001 (IR vs IR+miR-486-5p) (b) Graph shows kidney protein expression of eNOS, with representative immunoblot. Protein expression was normalized to GAPDH. *p<0.05 sham vs IR; n=3 rats per group for sham and IR+miR-486-5p groups, and n=4 for IR group

Supplementary figure 7

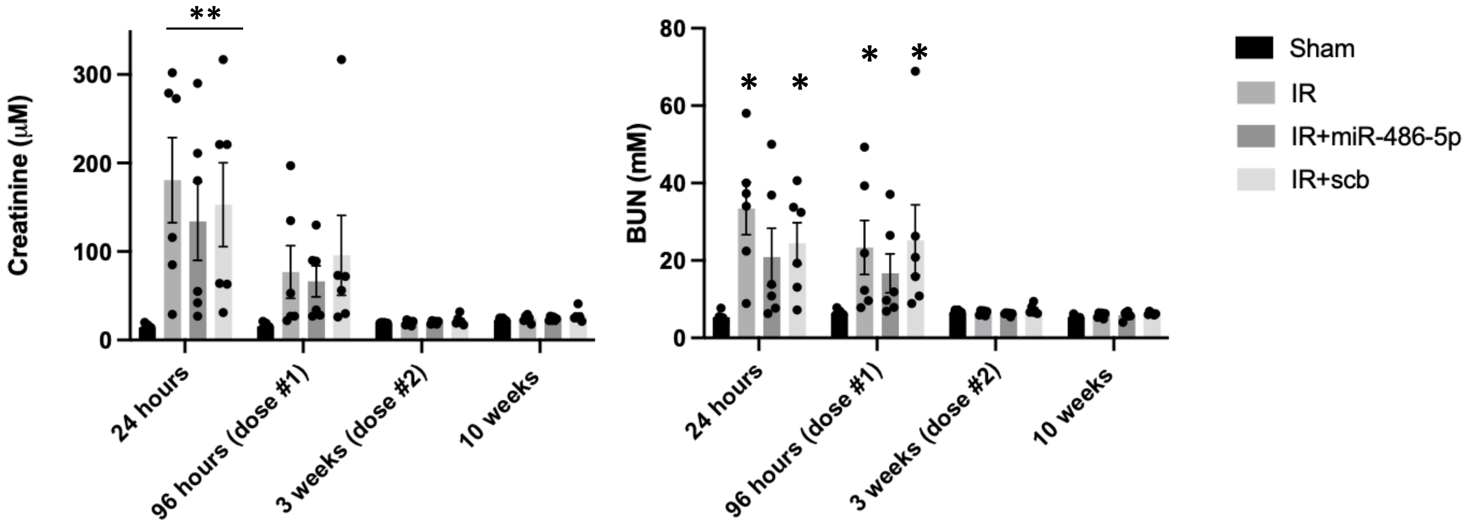

Figure S8. Effect of delayed miR-486-5p administration on kidney function after ischemia-reperfusion (IR) injury. Kidney function by plasma creatinine (Cr) and BUN levels with late administration of miR-486-5p or scramble (scb) miRNA at 96 h and 3 weeks after reperfusion. Plasma Cr and BUN are reported in standard SI units. To convert plasma Cr from μM to mg/dL, multiply by 0.0113, and to convert mM BUN to mg/dL, multiply by 2.8. **p<0.01, *p<0.05 vs sham; n=6 rats per group.

Supplementary figure 8

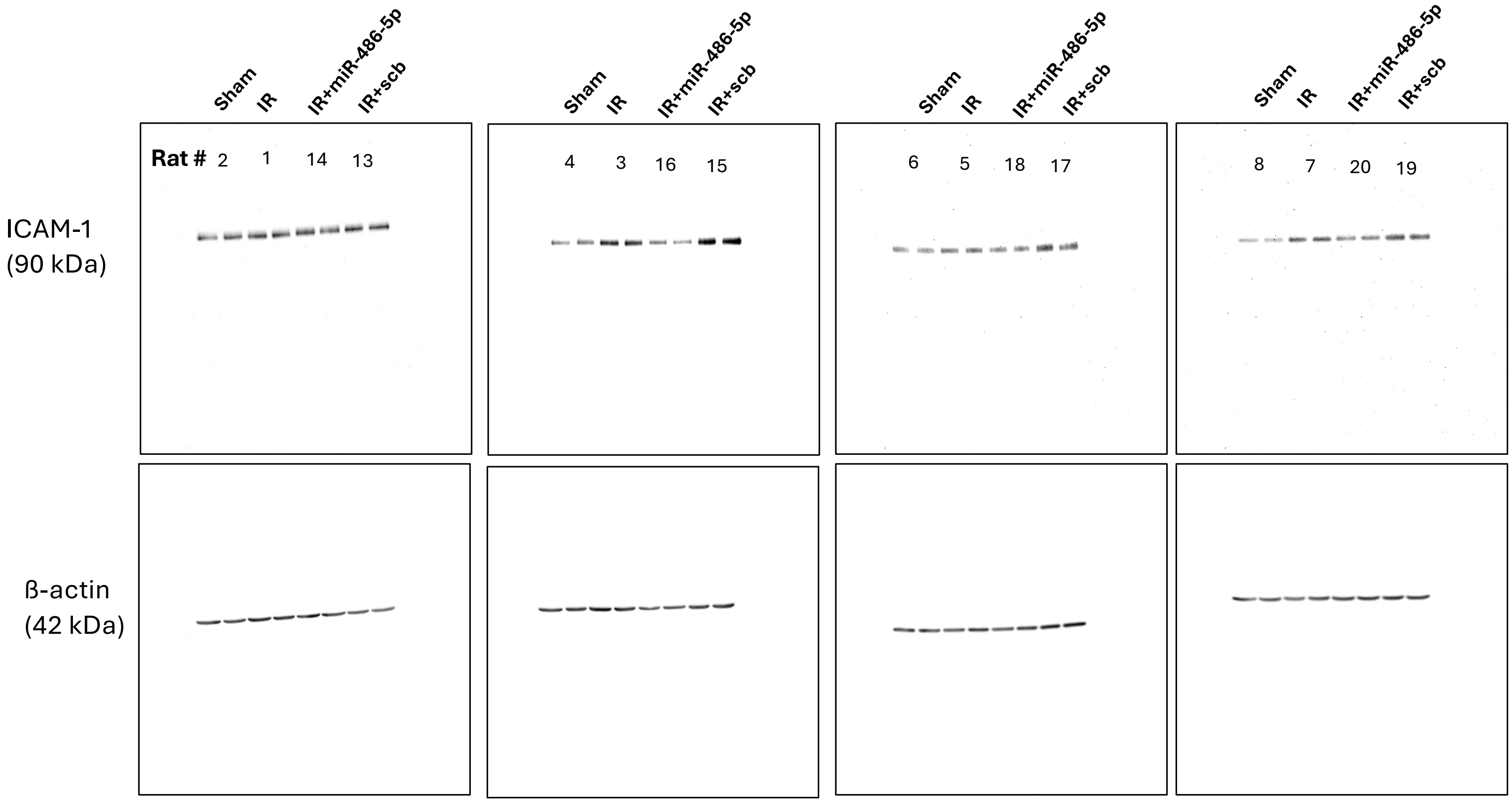

Immunoblots of ICAM-1 and ß-actin in rat kidney 24 hrs after kidney ischemia-reperfusion (IR) injury with administration of miR-486-5p i.v. at the start of reperfusion. Shown are the immunoblots for 16 rats (numbered at the top). Technical duplicates are loaded. (Total N=7 rats per group)

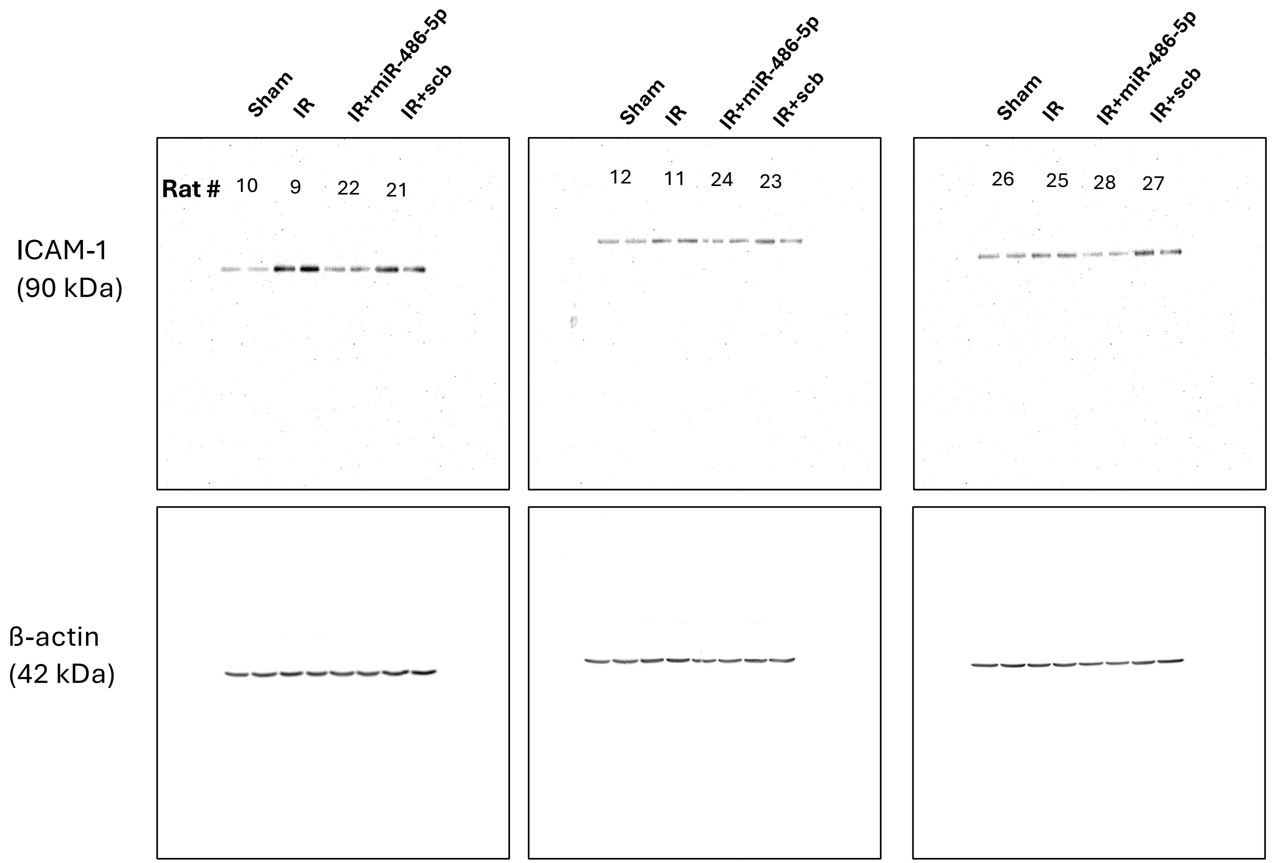

Immunoblots of ICAM-1 and ß-actin in rat kidney 24 hrs after kidney ischemia-reperfusion (IR) injury with administration of miR-486-5p i.v. at the start of reperfusion. Shown are the immunoblots for the next 12 rats (numbered at the top). Technical duplicates are loaded. (Total N=7 rats per group)

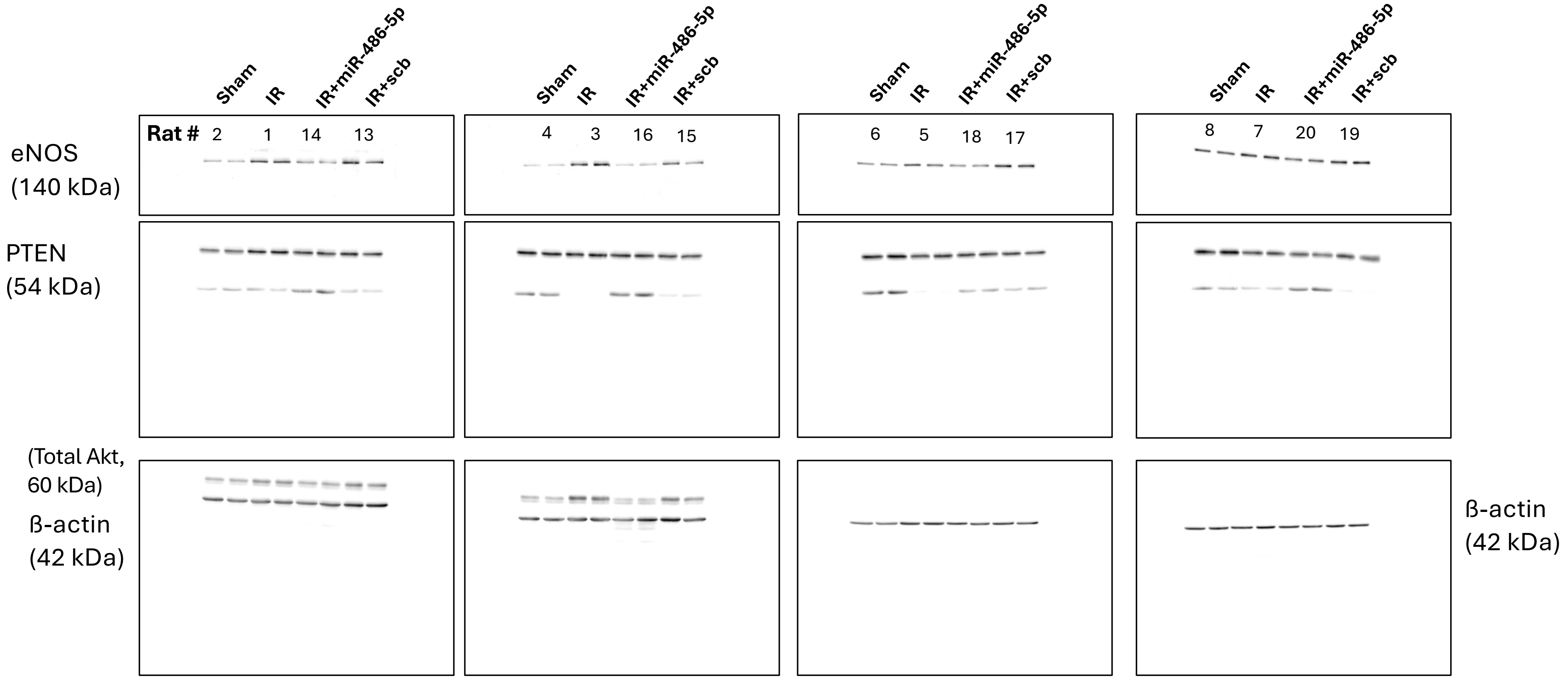

Immunoblots of eNOS, PTEN and ß-actin in rat kidney 24 hrs after kidney ischemia-reperfusion (IR) injury with administration of miR-486-5p i.v. at the start of reperfusion. Shown are the immunoblots for 16 rats (numbered at the top). Technical duplicates are loaded.
Total Akt is not included in the manuscript. (Total N=7 rats per group)

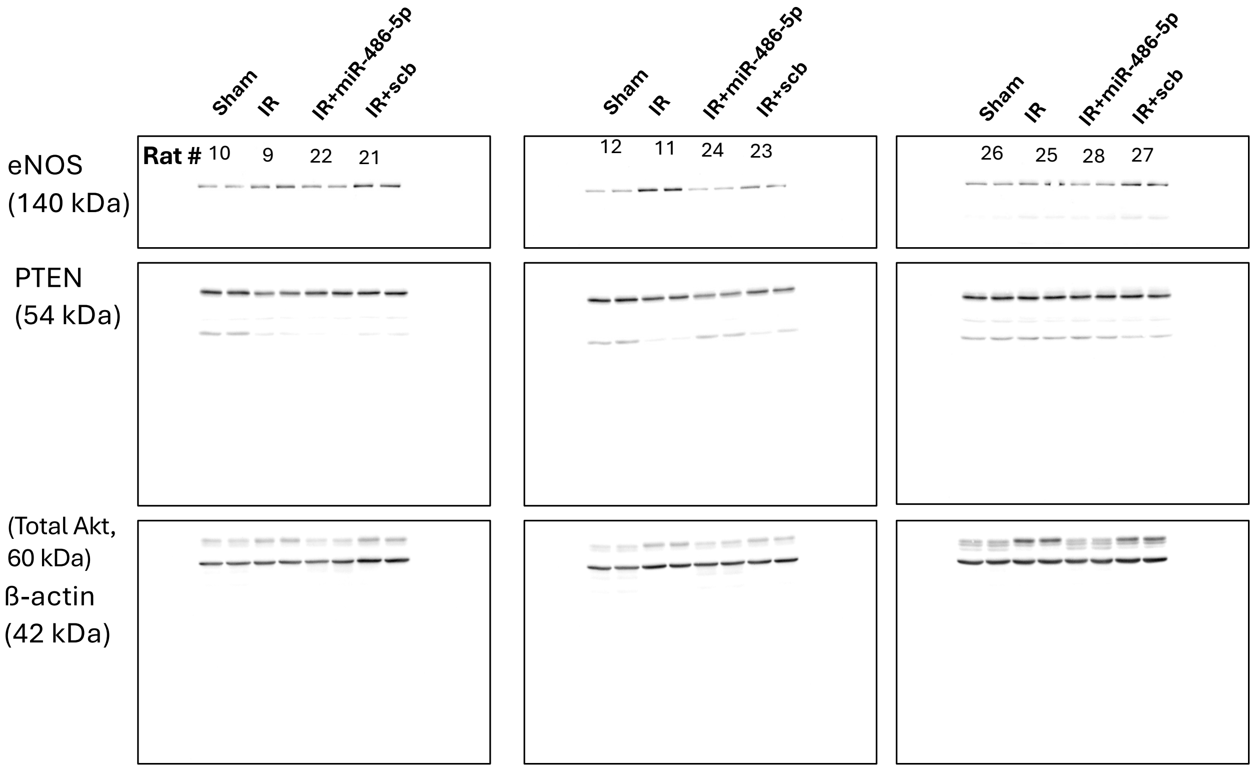

Immunoblots of eNOS, PTEN and ß-actin in rat kidney 24 hrs after kidney ischemia-reperfusion (IR) injury with administration of miR-486-5p i.v. at the start of reperfusion. Shown are the immunoblots for the next 12 rats (numbered at the top). Technical duplicates are loaded.
Total Akt is not included in the manuscript. (Total N=7 rats per group)

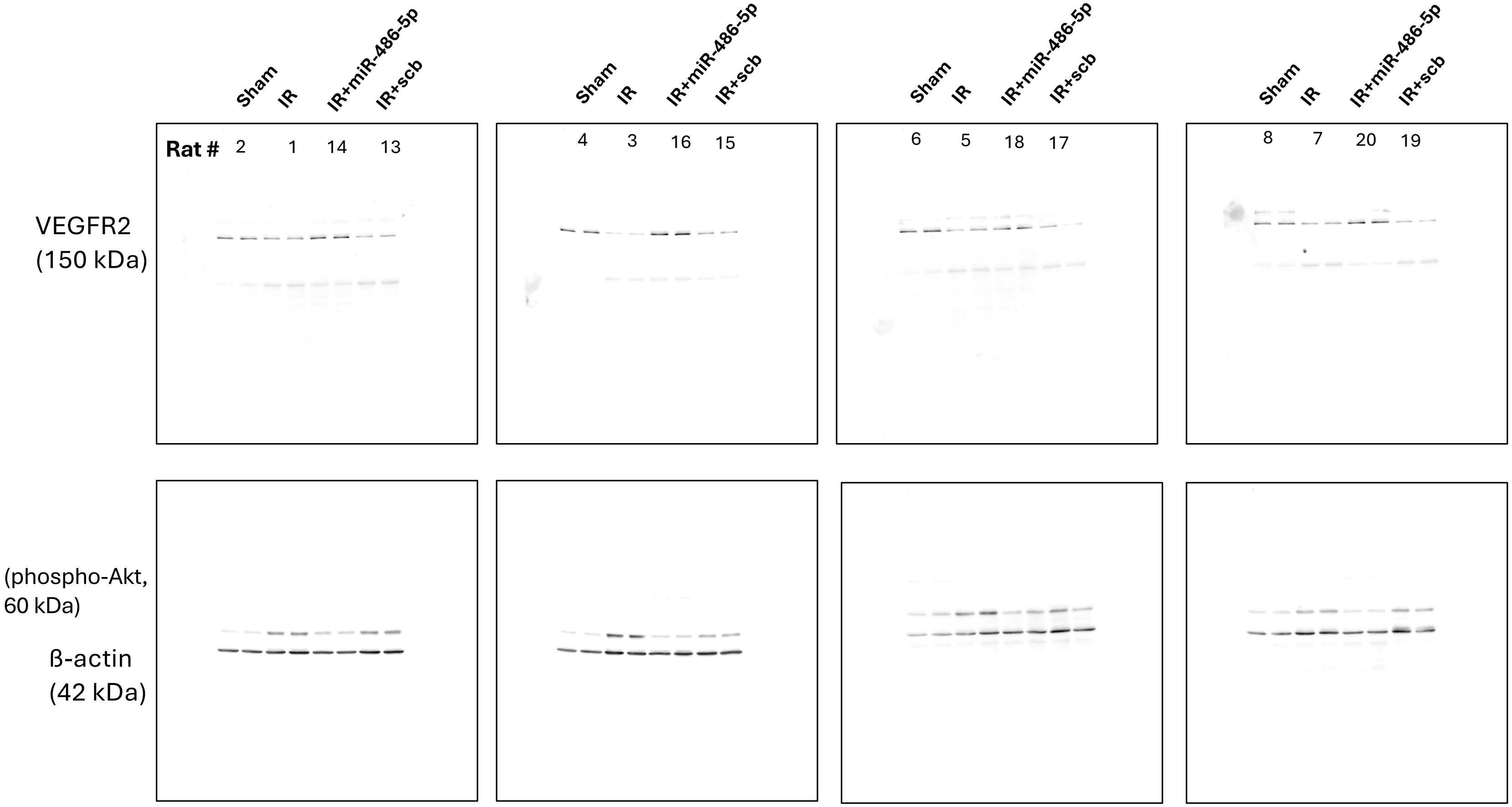

Immunoblots of VEGFR2 and ß-actin in rat kidney 24 hrs after kidney ischemia-reperfusion (IR) injury with administration of miR-486-5p i.v. at the start of reperfusion. Shown are the immunoblots for 16 rats (numbered at the top). Technical duplicates are loaded. (Total N=7 rats per group)

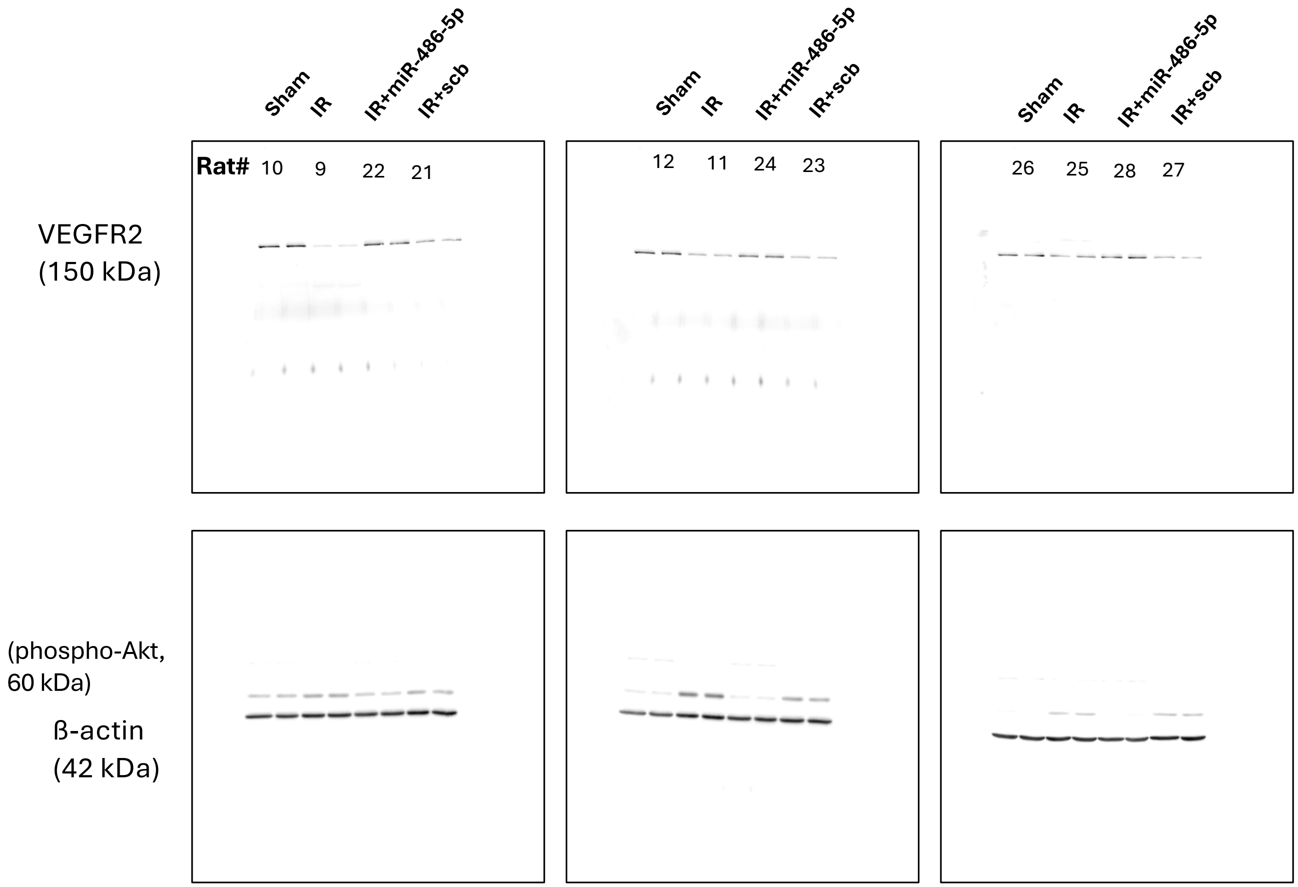

Immunoblots of VEGFR2 and ß-actin in rat kidney 24 hrs after kidney ischemia-reperfusion (IR) injury with administration of miR-486-5p i.v. at the start of reperfusion. Shown are the immunoblots for the next 12 rats (numbered at the top). Technical duplicates are loaded. Phospho-Akt is not included in the manuscript. (Total N=7 rats per group)

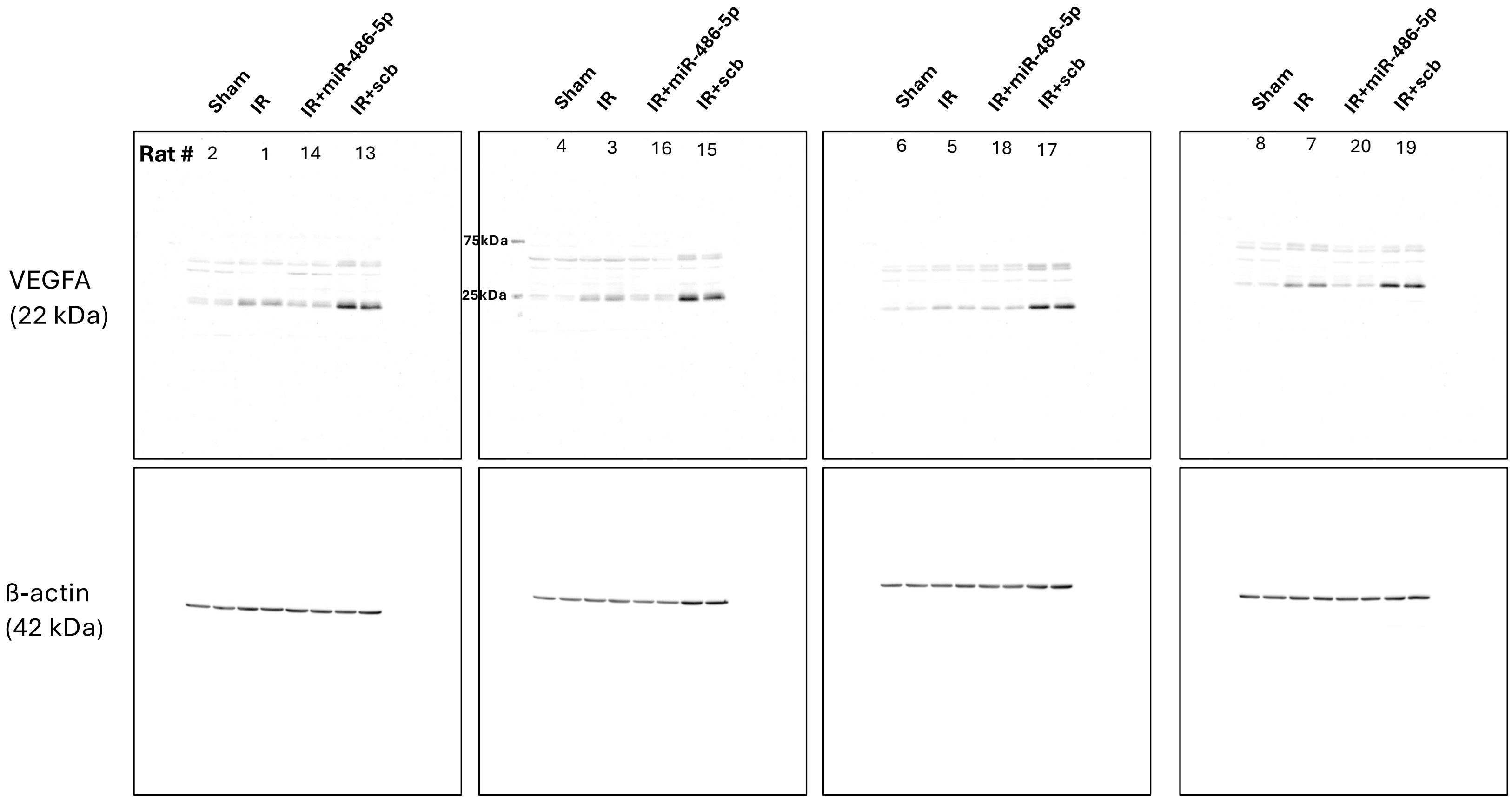

Immunoblots of VEGFA and ß-actin in rat kidney 24 hrs after kidney ischemia-reperfusion (IR) injury with administration of miR-486-5p i.v. at the start of reperfusion. Shown are the immunoblots for 16 rats (numbered at the top). The VEGFA monomer was detected at 22 kDa. Technical duplicates are loaded. (Total N=7 rats per group)

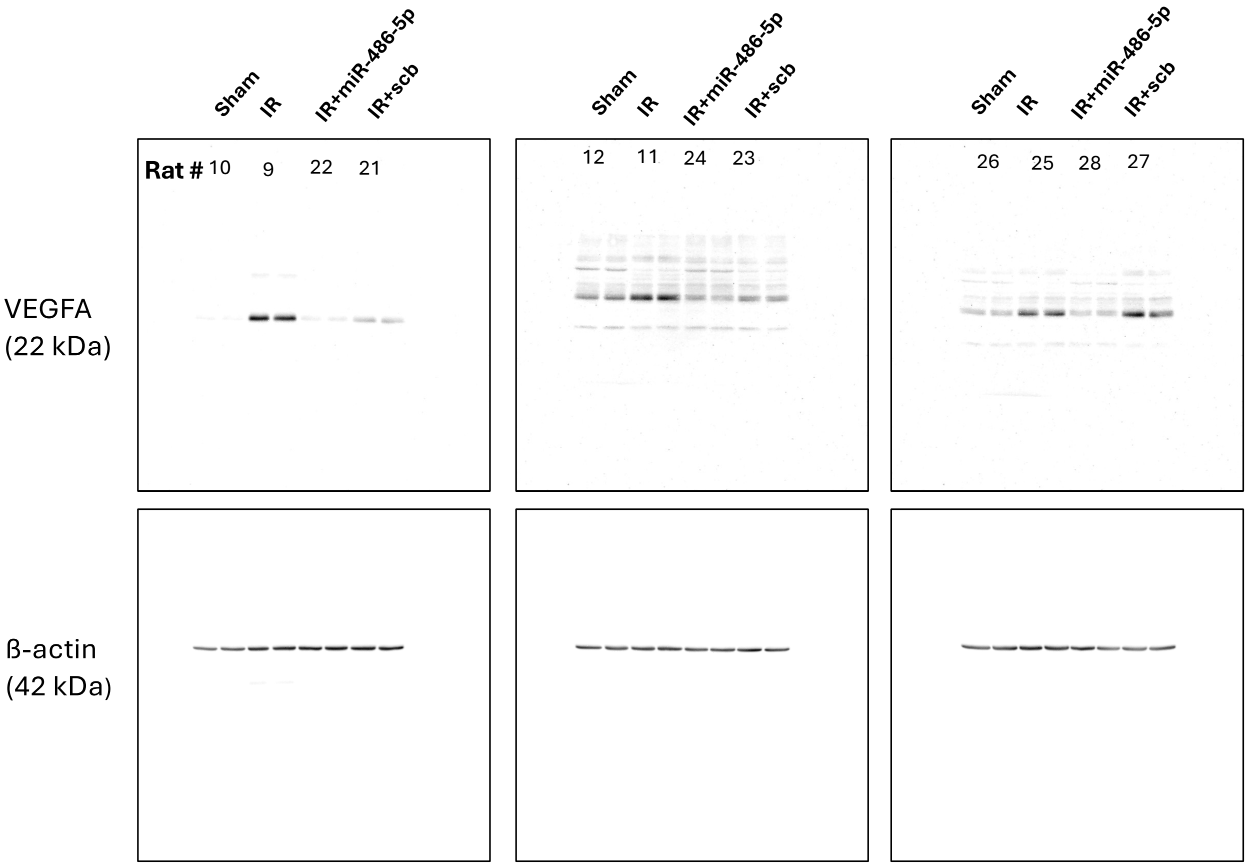

Immunoblots of VEGFA and ß-actin in rat kidney 24 hrs after kidney ischemia-reperfusion (IR) injury with administration of miR-486-5p i.v. at the start of reperfusion. Shown are the immunoblots for the next 12 rats (numbered at the top). Technical duplicates are loaded. (Total N=7 rats per group)

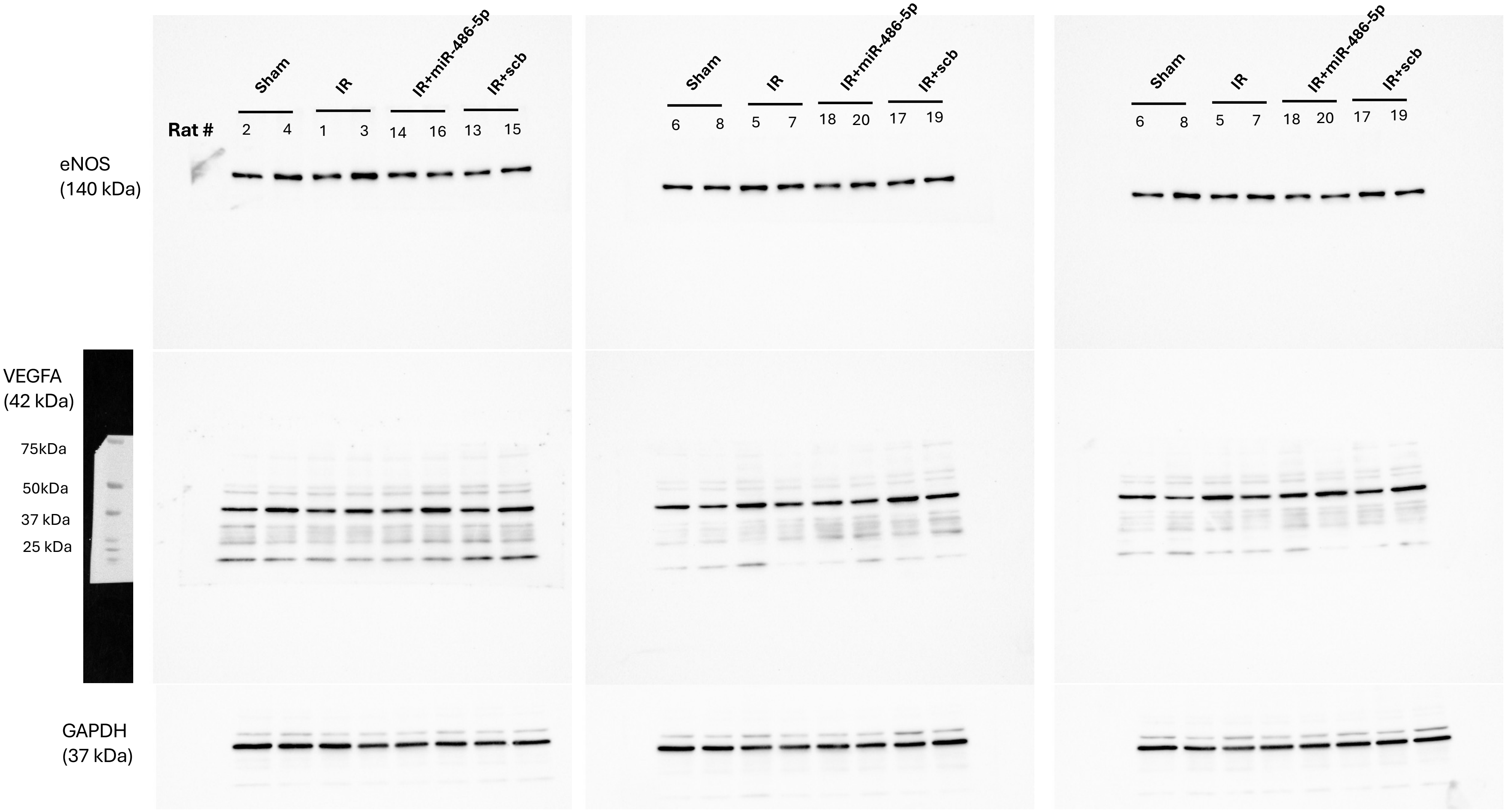

Immunoblots of eNOS, VEGFA, and GAPDH in rat liver 24 hrs after kidney ischemia-reperfusion (IR) injury with administration of miR-486-5p i.v. at the start of reperfusion. The VEGFA dimer was detected at 42 kDa. N=6 rats per group.

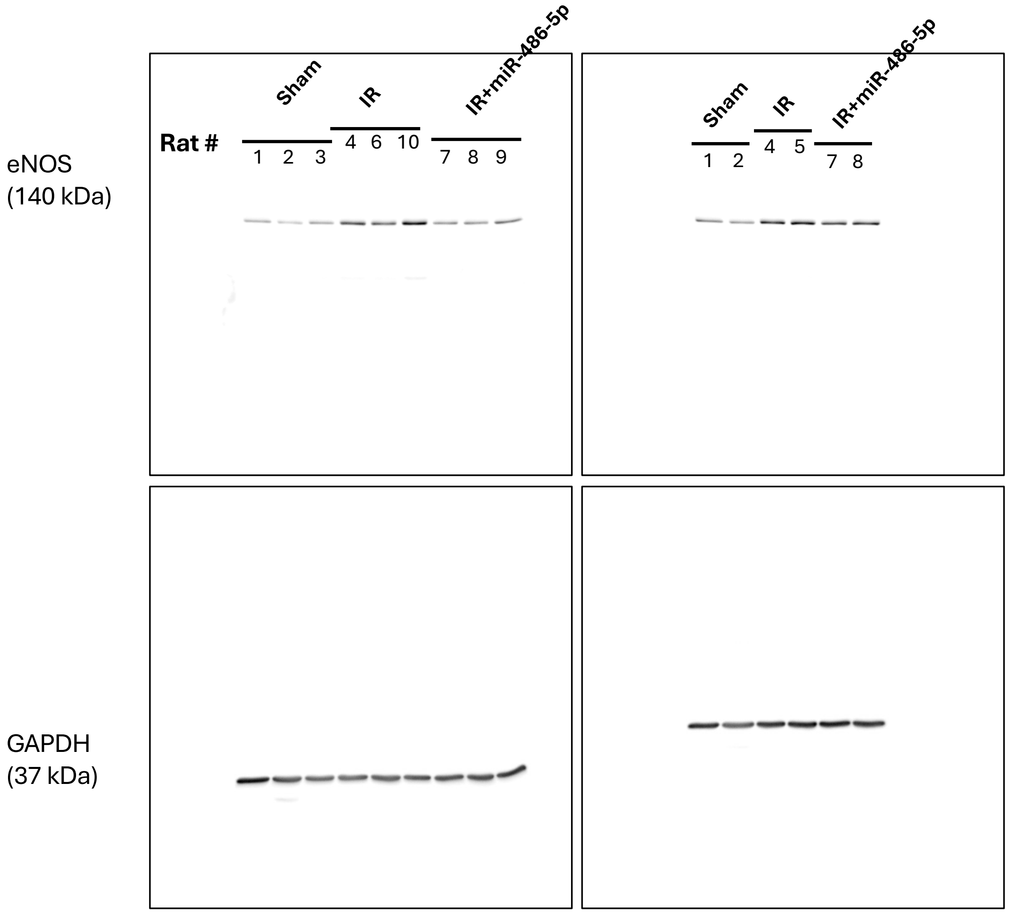

Immunoblots of eNOS and GAPDH in rat kidney 120 hrs after kidney ischemia-reperfusion (IR) injury with late administration of miR-486-5p after 96 hrs of reperfusion. A separate group of rats was used for this analysis. Groups include sham-operated rats (n=3, rats #1-3), rats with kidney IR injury alone (n=4, rats # 4-6, 10) and rats with kidney IR injury with administration of miR-486-5p after 96 hrs (n=3, rats #7-9).

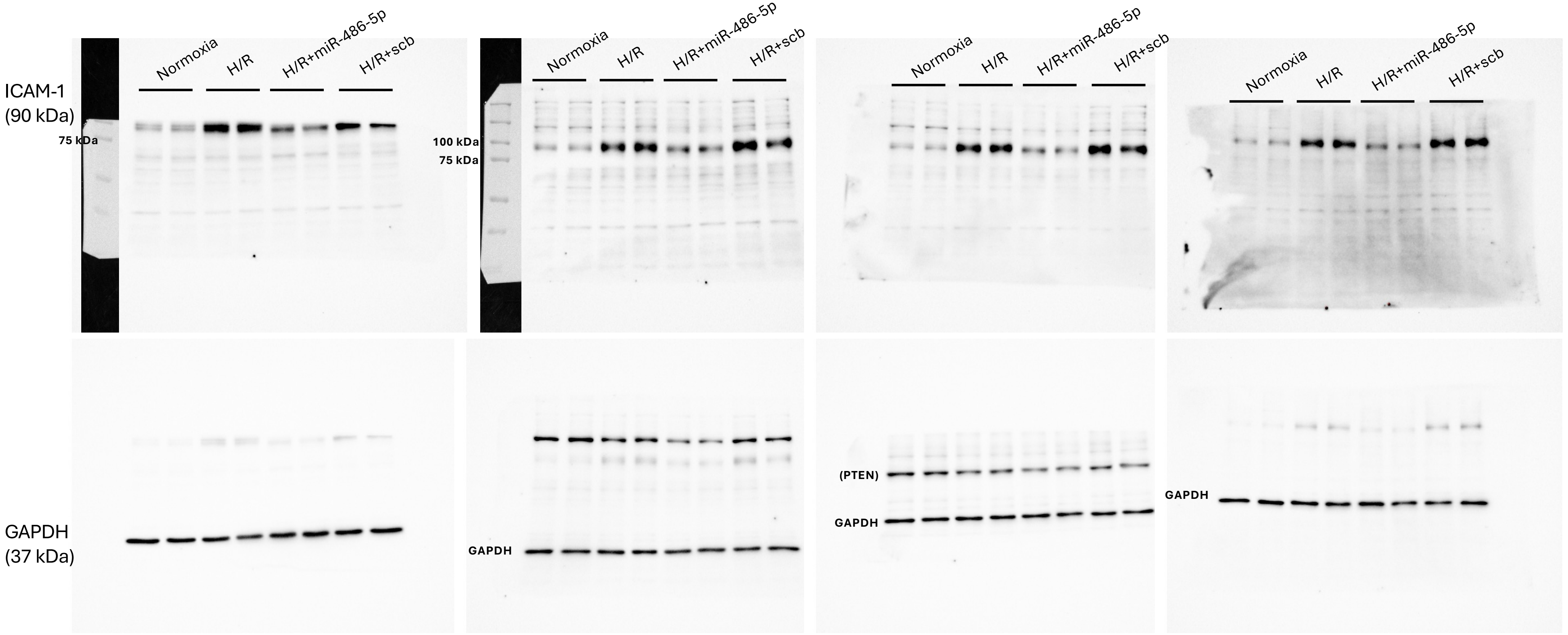

Immunoblots for ICAM-1 and GAPDH in human umbilical vein endothelial cells (HUVECs) transfected with miR-486-5p mimic (1nM) or scramble miRNA (1nM) and subjected to hypoxia-reoxygenation (H/R). Technical duplicates are loaded. PTEN in HUVECs is not included in the manuscript. N=4 experiments.
